# Ficolin B secreted by alveolar macrophage exosomes exacerbates bleomycin-induced lung injury via ferroptosis through the cGAS-STING signaling pathway

**DOI:** 10.1038/s41419-023-06104-4

**Published:** 2023-08-30

**Authors:** Xu Wu, Yixia Jiang, Rong Li, Yezhou Xia, Feifan Li, Meiyun Zhao, Guoqing Li, Xiaowu Tan

**Affiliations:** 1 Pulmonary and Critical Care Medicine, the Second Affiliated Hospital, Hengyang Medical School, University of South China, Hengyang, Hunan China; 2 Department of Gastroenterology, the Second Affiliated Hospital, Hengyang Medical School, University of South China, Hengyang, Hunan China; 3 The Key Laboratory of Molecular Diagnosis and Precision Medicine in Hengyang, Hengyang, Hunan China; 4 The Clinical Research Center for Gastric Cancer in Hunan Province, Hengyang, Hunan China

**Keywords:** Immunology, Cell death

## Abstract

Pathogenesis exploration and timely intervention of lung injury is quite necessary as it has harmed human health worldwide for years. Ficolin B (Fcn B) is a recognition molecule that can recognize a variety of ligands and play an important role in mediating the cell cycle, immune response, and tissue homeostasis in the lung. However, the role of Fcn B in bleomycin (BLM)-induced lung injury is obscure. This study aims to investigate the sources of Fcn B and its mechanism in BLM-induced lung injury. WT, *Fcna*^*-/-*^, and *Fcnb*^*-/-*^ mice were selected to construct the BLM-induced lung injury model. Lung epithelial cells were utilized to construct the BLM-induced cell model. Exosomes that were secreted from alveolar macrophages (AMs) were applied for intervention by transporting Fcn B. Clinical data suggested M-ficolin (homologous of Fcn B) was raised in plasma of interstitial lung disease (ILD) patients. In the mouse model, macrophage-derived Fcn B aggravated BLM-induced lung injury and fibrosis. Fcn B further promoted the development of autophagy and ferroptosis. Remarkably, cell experiment results revealed that Fcn B transported by BLM-induced AMs exosomes accelerated autophagy and ferroptosis in lung epithelial cells through the activation of the cGAS-STING pathway. In contrast, the application of 3-Methyladenine (3-MA) reversed the promotion effect of Fcn B from BLM-induced AMs exosomes on lung epithelial cell damage by inhibiting autophagy-dependent ferroptosis. Meanwhile, in the BLM-induced mice model, the intervention of Fcn B secreted from BLM-induced AMs exosomes facilitated lung injury and fibrosis *via* ferroptosis. In summary, this study demonstrated that Fcn B transported by exosomes from AMs exacerbated BLM-induced lung injury by promoting lung epithelial cells ferroptosis through the cGAS-STING signaling pathway.

## Introduction

The lung is an important part of the respiratory system and is susceptible to damage from the organism and external stimuli [1]. Lung injury is one of the common diseases clinically, a result of exposure to various stimulatory factors such as hypoxia, reperfusion, and xenobiotics [2]. It is characterized by diffuse alveolar injury, which can lead to lung inflammation and death of lung epithelial cells [3]. With the improvement of pathophysiology understanding and treatment techniques, new therapies such as extracorporeal membrane oxygenation and lung protective ventilation have been developed. However, the pathogenesis of lung injury involved in multi-stage, multi-level, and multi-pathway has not been fully elucidated, and the mortality rate of lung injury is still high [4]. Therefore, it is urgent to further explore the pathogenesis of lung injury and develop effective strategies.

Ferroptosis, a kind of programmed cell death that is different from necrotic apoptosis, depends on iron and reactive oxygen species (ROS) [5]. The related mechanisms of ferroptosis mainly include iron metabolism [6], the Nrf2/GPX4 pathway [7], and cystine/glutamate antiporter [8]. It has been reported that ferroptosis is closely involved in the occurrence and development of lung injury and pulmonary fibrosis [9]. Iron accumulation in the lower respiratory tract could lead to inflammatory responses, oxidative stress, and mitochondrial dysfunction, aggravating lung injury [10]. Liu et al. demonstrated the involvement of ferroptosis in the progression of lipopolysaccharide (LPS)-induced lung injury using ferrostatin-1, an inhibitor of ferroptosis [11]. Likewise, regulation of autophagy activity can improve LPS-induced lung injury [12, 13]. Recent studies have shown that some proteins and signaling pathways regulating autophagy are also involved in the occurrence of ferroptosis, affecting the process of cell death [14]. Therefore, ferroptosis is a type of autophagy-dependent cell death in some diseases, and its key regulatory molecules include ATG7, Beclin1, SLC7A11, and so on [15]. Knockout of ATG7 can inhibit ferroptosis by reducing intracellular Fe^2+^ levels and lipid peroxidation [16]. Beclin1 can promote ferroptosis by binding directly to SLC7A11 [17]. As a kind of selective autophagy, ferritinophagy, with nuclear receptor coactivator 4 (NCOA4) as the cargo receptor protein, can also regulate ferroptosis in cells by degrading ferritins [18]. Therefore, it is a good therapeutic strategy to relieve lung injury by regulating autophagy and ferroptosis simultaneously.

Ficolins, novel pattern recognition molecules of the innate immune system, play an important role in lung diseases due to their sustained high expression in the lungs, recognition of a variety of ligands, and other characteristics [19]. Rodents and most other mammals produce Ficolin A (Fcn A, Ficolin-2) and Ficolin B (Fcn B, Ficolin-1), homologs of L-ficolin and M-ficolin, respectively [20]. The structure of M-ficolin is 76% identical to L-ficolin [21]. M-ficolin is secreted by monocytes/macrophages and can be circulated in human plasma [22]. In lung disease studies, M-ficolin has been shown to modulate the immune response of lung epithelial cells induced by fungal polysaccharides [23]. Other studies have suggested that Fcn B may play a role in clearing apoptotic and necrotic cells and maintaining tissue homeostasis [24]. In addition, the correlation between serum Ficolin-1 levels and ferritin expression in patients with autoimmune diseases has already been investigated [25]. Thus, we speculate that Fcn B may have the potential to mediate cell death and iron metabolism to regulate lung diseases. In previous studies, we showed that Fcn A played a marked role in LPS or H1N1 virus-induced acute lung injury. However, Fcn B had no significant role in these two models of lung injury [26, 27]. In clinics, bleomycin (BLM) is often used as an anticancer drug, but it has serious side effects, of which pulmonary toxicity is the most significant [28]. Moreover, the BLM-induced lung injury model can better reflect the pathological characteristics of patients with pulmonary fibrosis, so it is often used in research on lung diseases [29]. However, there is no report on the application study of Fcn B in BLM-induced lung injury.

Research has shown that the cyclic GMP-AMP synthase-stimulator of interferon genes (cGAS-STING) pathway is the cause of various inflammatory and autoimmune diseases [30]. Recently, there has been evidence that overactivation of the cGAS-STING pathway can drive lung diseases, making it a promising therapeutic target for inflammatory lung disease [31]. A study suggests that activating STING drives silica-induced lung inflammation [32]. In addition, activating the cGAS pathway and inhibiting endothelial cell proliferation promotes inflammatory lung injury [33]. Other studies have shown that airway epithelial cGAS is crucial for inducing experimental allergic airway inflammation [34]. In the study of lung ischemia/reperfusion injury, inhibition of the cGAS-STING pathway alleviates the degree of injury by regulating Endoplasmic reticulum stress of rat alveolar epithelial type II cells [35]. In recent studies, the cGAS-STING pathway has been involved in regulating inflammatory lung injury mediated by neutral extracellular traps (NETs) [36]. In addition, downregulating the STING pathway inhibits inflammatory response and oxidative stress, thereby alleviating LPS-induced acute lung injury (ALI) [37]. However, there have been no reports on whether the cGAS-STING pathway mediates the role of Fcn B in BLM-induced lung injury.

Here, we applied WT, *Fcna*^*-/-*^ and *Fcnb*^*-/-*^ mice to construct BLM-induced lung injury models, and mouse lung epithelial cells to construct a BLM-induced cell model to explore the sources of Fcn B and its mechanism in lung injury, which enriched insights into the pathogenesis of lung injury.

## Materials and methods

### Construction of BLM-induced lung injury model and intervention

Female WT, *Fcna*^*-/-*^, and *Fcnb*^*-/-*^ mice (6–10 weeks) were used in this study as described before [26]. After 1 week of adaptive feeding, the BLM-induced lung injury model was constructed [38]. Mice in experiment 1 were divided into the following groups: WT, *Fcna*^*-/-*^, *Fcnb*^*-/-*^, WT+BLM, *Fcna*^*-/-*^+BLM, *Fcnb*^*-/-*^+BLM, 10 mice in each group. Mice were anesthetized by intraperitoneal injection of 3% sodium pentobarbital and fixed on the operating plate. Mice in WT+BLM, *Fcna*^*-/-*^+BLM, and *Fcnb*^*-/-*^+BLM groups were injected intratracheally with BLM (2.5 mg/kg, 100 μL/20 g) (B107423, Aladin, China) using a syringe. Mice in WT, *Fcna*^*-/-*^, and *Fcnb*^*-/-*^ groups were injected with the same volume of normal saline into the trachea. After injection, the mice were rotated upright for 2 min so that BLM or normal saline was evenly distributed in both lungs. The wound was sutured, and the mice were routinely fed after they recovered naturally.

Mice in experiment 2 were divided into the following groups: WT, *Fcnb*^-/-^, WT+BLM, WT+BLM+Exo^NC^, WT+BLM+Exo^oe-FcnB^, *Fcnb*^-/-^+BLM, *Fcn*^-/-^+BLM+Exo^NC^, *Fcnb*^-/-^+BLM+Exo^oe-Fcnb^, 10 mice in each group. BLM-induced lung injury models were constructed as in experiment 1. Mice in WT+BLM+Exo^NC^ and *Fcnb*^-/-^+BLM+Exo^NC^ groups were simultaneously injected with exosomes (100 μL, 1 g/kg) derived from oe-NC plasmids-transfected and BLM-treated MH-S through the tail vein. Mice in WT+BLM+Exo^oe-Fcnb^ and *Fcnb*^-/-^+BLM+Exo^oe-Fcnb^ groups were simultaneously injected with exosomes (100 μL, 1 g/kg) derived from oe-Fcnb plasmids-transfected and BLM-treated MH-S through the tail vein. Mice in WT, *Fcnb*^*-/-*^, and WT+BLM groups were simultaneously injected with the same amount of normal saline through the tail vein. The intervention was performed weekly for 3 weeks. Whole blood samples of mice were obtained by eyeball blood collection after anesthesia. Alveolar lavage fluid was collected after the mice were sacrificed for cervical dislocation. Lung tissue samples were obtained from mice dissected under sterile conditions. Mitochondria damage and the number of autophagosomes of lung tissues in the above groups were observed by transmission electron microscopy (TEM, EM1400, JEOL, Japan). All procedures were approved by the Biomedical Research Ethics Committee of the University of South China (NHFE2022010601).

### Cell culture and treatment

Mouse lung epithelial cells (MLE-12, AW-CNM486, Abiowell, China) were cultured in DMEM/F-12 (D8437, Sigma, USA) containing 10% fetal bovine serum (FBS, 10099141, Gibco, USA) and 1% Penicillin/Streptomycin (AWH0529b, Abiowell, China). Mouse alveolar macrophages MH-S (AW-CNM405, Abiowell, China) were cultured in 1640 (R8758, Sigma, USA) containing 10% FBS and 1% Penicillin/Streptomycin. Cells were placed in a humidified incubator containing 5% CO_2_ at 37 °C. When the cell confluence reached 70–80%, trypsin digestion, passage, and transfection experiments were carried out.

In our study, we used the Transwell chamber system for co-culture experiments. The Transwell chamber system consisted of an upper and a lower chamber separated by a porous membrane that allowed the passage of exosomes while preventing direct cell-to-cell contact. AMs were inoculated in the upper chamber and MLE-12 cells in the lower chamber at a density of 1 × 10^4^ cells. BLM can be added to the upper chamber and the lower chamber, respectively, to induce the cells. In the intervention experiment, GW4869 was added to the upper chamber to inhibit the release of exosomes by AMs. The Transwell chamber system was then placed in a humidified incubator containing 5% CO_2_ at 37 °C for 24 h. Finally, MLE-12 cells in the lower chamber were isolated for subsequent analysis

Cells in Experiment 1 were divided into the following groups: Control, BLM, BLM+co-AMs, BLM+co-BLM-AMs, and BLM+co-BLM-AMs+GW4869. In the Control group, MLE-12 was cultured normally. In the BLM group, MLE-12 was treated with 40 μg/mL BLM for 24 h. In the BLM+co-AMs group, MLE-12 was treated with 40 μg/mL BLM for 24 h and co-cultured with MH-S for another 24 h. In the BLM+co-BLM-AMs group, MLE-12 and MH-S were both treated with 40 μg/mL BLM for 24 h, and two kinds of cells were co-cultured for 24 h. In the BLM+co-BLM-AMs+GW4869 group, MLE-12 and MH-S were both treated with 40 μg/mL BLM for 24 h and then co-cultured for 24 h after the addition of 10 μM GW4869 (D1692, Sigma, USA) [39].

Cells in Experiment 2 were divided into the following groups: Control, BLM, BLM+BLM-Exo, BLM^si-NC^, BLM^si-Fcnb^, BLM^si-Fcnb^+Exo^oe-NC^, and BLM^si-Fcnb^+Exo^oe-Fcnb^. In the Control group, MLE-12 was cultured normally. In the BLM group, MLE-12 was treated with 40 μg/mL BLM for 24 h. In the BLM+BLM-Exo group, MLE-12 were treated with 40 μg/mL BLM for 24 h and then co-cultured with exosomes derived from MH-S that were treated with 40 μg/mL BLM for another 24 h. In BLM^si-NC^ and BLM^si-Fcnb^ groups, MLE-12 were transfected with si-NC or si-Fcnb plasmids and then treated with 40 μg/mL BLM for 24 h. In BLM^si-Fcnb^+Exo^oe-NC^ and BLM^si-Fcnb^+Exo^oe-Fcnb^ groups, MLE-12 were transfected with si-Fcnb plasmids and treated with 40 μg/mL BLM for 24 h, and then treated with exosomes derived from oe-NC or oe-Fcnb plasmids-transfected and BLM-treated MH-S for 24 h.

Cells in Experiment 3 were divided into the following groups: Control, BLM^si-NC^, BLM^si-cGAS^, BLM^si-cGAS^+Exo^oe-NC^, and BLM^si-cGAS^+Exo^oe-Fcnb^. In the Control group, MLE-12 was cultured normally. In BLM^si-NC^ and BLM^si-Fcnb^ groups, MLE-12 were transfected with si-NC or si-Fcnb plasmids and then treated with 40 μg/mL BLM for 24 h. In BLM^si-cGAS^+Exo^oe-NC^ and BLM^si-cGAS^+Exo^oe-Fcnb^ groups, MLE-12 were transfected with si-cGAS plasmids and treated with 40 μg/mL BLM for 24 h, and then treated with exosomes derived from oe-NC or oe-Fcnb plasmids-transfected and BLM-treated MH-S for another 24 h.

Cells in Experiment 4 were divided into the following groups: BLM^si-NC^, BLM^si-cGAS^, BLM^si-cGAS^+Exo^oe-NC^, BLM^si-cGAS^+Exo^oe-Fcnb^, 3-MA, and BLM^si-cGAS^+Exo^oe-Fcnb^+3-MA. In BLM^si-NC^ and BLM^si-cGAS^ groups, MLE-12 were transfected with si-NC or si-cGAS plasmids and then treated with 40 μg/mL BLM for 24 h. In BLM^si-cGAS^+Exo^oe-NC^ and BLM^si-cGAS^+Exo^oe-Fcnb^ groups, MLE-12 were transfected with si-cGAS plasmids, treated with 40 μg/mL BLM for 24 h, and then treated with exosomes derived from oe-NC or oe-Fcnb plasmids-transfected and BLM-treated MH-S. In the 3-MA group, MLE-12 were treated with 5 mM 3-Methyladenine (3-MA) for 24 h. In the BLM^si-cGAS^+Exo^oe-Fcnb^+3-MA group, MLE-12 were transfected with si-cGAS plasmids, treated with 40 μg/mL BLM for 24 h, followed by 5 mM 3-MA for 24 h, and then treated with exosomes derived from oe-Fcnb plasmids-transfected and BLM-treated MH-S. Mitochondria damage and the number of autophagosomes in the above groups were observed by TEM.

### Extraction and identification of exosomes

According to the instructions of the exosome extraction kit (EXOTC10A-1, SBI, USA), exosomes were extracted from the supernatant of AMs culture. The extracted exosomes were suspended with PBS for subsequent identification experiments. TEM was employed to characterize the morphology of exosomes. Nanoparticle tracking analysis (NTA, ZetaView PMX 110, Particle Matrix, Germany) was performed to characterize the particle size of exosomes. Western blot was used to detect the expressions of biomarkers CD9, CD63, and CD81 in exosomes. Exosomes were labeled with PKH67 and co-cultured with MLE-12 to observe the uptake of exosomes by MLE-12.

### Hematoxylin-eosin (HE) staining

HE staining was utilized to detect the lung histopathological changes of mice in each group. After the lung tissues were washed with normal saline, they were fixed with a 4% paraformaldehyde solution and then sectioned for use after paraffin embedding. First, the slices were dewaxed by placing them in xylene for 20 min. Then dehydration was carried out with gradient ethanol (75–100%), 5 min for each level. The slices were stained with hematoxylin (AWI0001a, Abiowell, China) for 1 min, rinsed with distilled water, and then returned to blue in PBS. Next, the slices were stained with eosin (AWI0029a, Abiowell, China) for 1 min and rinsed with distilled water. Dehydration was carried out with gradient alcohol (95–100%), 5 min for each level. The slices were cleared in xylene for 10 min and then sealed with neutral gum (AWI0238a, Abiowell, China) for observation by microscope.

### Masson staining

Masson staining (AWI0253, Abiowell, China) was used to detect pulmonary interstitial fibrosis of mice in each group. First, the lung slices were dewaxed to water. Hematoxylin stain solution was added to cover the slices and then stained for 1 min. The slices were washed with tap water and distilled water in turn. Then, the slices were soaked in PBS (pH 7.2–7.6) or ammonia for 10 min to make the nucleus return blue. Acid fuchsin stain solution was added and stained for 5 min. After that, the slices were reacted with a phosphomolybdic acid differentiation solution for about 30 s. The tissue was covered by a drop of aniline blue counterstain, stained for 1 min, and rinsed with absolute ethanol. The slices were blow-dried, cleared in xylene, and then sealed with neutral gum for observation by microscope.

### Biochemical detection

The concentrations of total protein in bronchoalveolar lavage fluid (BALF) (AWB0104, Abiowell, China), hydroxyproline (CSB-E08839m, CUSABIO, China), malondialdehyde (MDA) (A003-1-2, NJJCBIO, China), glutathione (GSH) (A006-2-1, NJJCBIO, China), Fe^2+^ (TC1015, Leagene, China), L-ficolin (Ab213778, Abcam, UK), and M-ficolin (Ab213777, Abcam, UK) were measured according to the instructions of kits. The death rate of lung epithelial cells was detected by a lactate dehydrogenase (LDH) assay kit (C0016, Beyotime, China).

### Western blot

Western blot was applied to detect the expressions of alpha-smooth muscle actin (α-SMA), microtubule-associated protein 1 light chain 3-II (LC3II), LC3I, Beclin1, p62, autophagy-related gene 7 (ATG7), glutathione peroxidase 4 (GPX4), solute carrier family 7 member 11 (SLC7A11), ferritin heavy chain 1 (FTH1), ferritin light chain 1 (FTL1), NCOA4 in MLE-12 or lung tissues, as well as CD9, CD81, CD63 in AMs-derived exosomes. First, different groups of samples were treated with RIPA lysate (AWB0136, Abiowell, China) to extract total proteins. The protein was separated by SDS-PAGE and transferred to a nitrocellulose (NC) membrane. Next, the NC membrane was soaked in 5% skimmed milk and blocked at room temperature for 1.5 h. The NC membrane was then incubated with the primary antibody at 4 °C overnight. Primary antibodies used in the experiment were as follows: α-SMA (1:2000, 55135-1-AP, Proteintech, USA), LC3 (1:1500, 14600-1-AP, Proteintech, USA), Beclin1 (1:1000, 11306-1-AP, Proteintech, USA), p62 (1:4000, 18420-1-AP, Proteintech, USA), ATG7 (1:1500, 10088-2-AP, Proteintech, USA), GPX4 (1:5000, 67763-1-Ig, Proteintech, USA), SLC7A11 (1:1000, 26864-1-AP, Proteintech, USA), FTH1 (1:1000, ab65080, Abcam, UK), FTL1 (1:5000, 10727-1-AP, Proteintech, USA), NCOA4 (1:2500, ab86707, Abcam, UK), CD9 (1:5000, 60232-1-Ig, Proteintech, USA), CD63 (1:300, 25682-1-AP, Proteintech, USA), CD81 (1:3000, 66866-1-Ig, Proteintech, USA), cGAS (1:1000, ab252416, Abcam, UK), STING (1:2000, 19851-1-AP, Proteintech, USA), p-STING (1:1000, PA5-105674, Thermo Scientific, USA), and β-actin (1:5000, 66009-1-Ig, Proteintech, USA). The NC membrane was incubated with HRP-goat anti-mouse IgG (1:5000, SA00001-1, Proteintech, USA) or HRP-goat anti-rabbit IgG (1:6000, SA00001-2, Proteintech, USA) at room temperature for 1.5 h. Finally, the NC membrane was incubated with ECL reagent (AWB0005, Abiowell, China) for 1 min and placed in the imaging system for analysis. β-actin was used as an internal reference protein, and the expression levels of each protein were analyzed using Quantity One 4.6.6 (Bio-Rad Inc., USA).

### Quantitative real-time polymerase chain reaction (RT-qPCR)

The mRNA expression of Fcnb in MLE-12 was detected by RT-qPCR. First, total RNA was extracted using the Trizol total RNA extraction kit (15596026, Thermo, USA), followed by the determination of concentration and purity. Then, an mRNA reverse transcription kit (CW2569, CWBIO, China) was used to reverse the transcription of mRNA into cDNA, and RT-qPCR was performed. Primers used in the experiment were as follows: Fcnb: F: GCTGGTGACTCTCTGACACC, R: GCTGGAAGTACTGCCGTCAT; β-actin: F: ACATCCGTAAAGACCTCTATGCC, R: TACTCCTGCTTGCTGATCCAC. Using β-actin as an internal reference gene, the relative mRNA expression of Fcnb was calculated by the 2^−^^△△Ct^ method.

### Flow cytometry

The expression level of Fcnb and the number of neutrophils and macrophages were detected by flow cytometry [40]. Lung tissue samples were digested to obtain cell suspension. Blood, BALF, and cell suspension were centrifugally stratified to obtain neutrophils and macrophages for re-suspension. Antibodies of LY-6G (11-9668-82, eBioscience, USA) and F4/80 (11-4801-82, eBioscience, USA) were added respectively to the suspensions of neutrophils and macrophages and incubated for 30 min away from light. Then, the mixture of Fcn B antibody (Ab70814, Abcam, UK) and Alexa Fluor 594-goat anti-rabbit IgG (A-11012, Invitrogen, USA) was added to the above cell suspensions and incubated for another 30 min away from light. After washing and suspension, the cells were detected by flow cytometry (A00-1-1102, Beckman, USA). In addition, ROS levels in lung tissues and MLE-12 were detected by flow cytometry. First, the tissue and cells were digested to obtain cell suspensions. The cell suspension was incubated with BODIPY™ 581/591 C11 fluorescent probe (A-11012, Invitrogen, USA) at 37 °C for 30 min and finally detected by flow cytometry.

### Data analysis

Data were analyzed using GraphPad Prism 8.0. All experimental data were expressed as mean ± standard deviation (SD). One-way analysis of variance (ANOVA) was used for comparison between groups.

## Results

### Macrophage-derived Fcn B aggravated BLM-induced lung injury and fibrosis

**Fig. 1:**
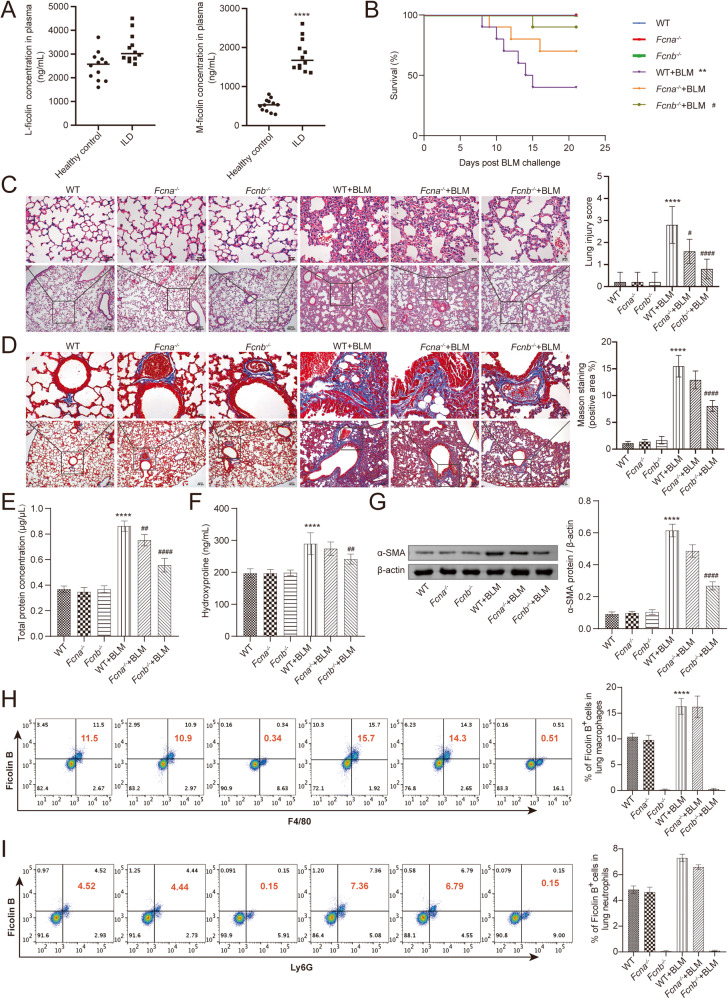
Fcn B from macrophages aggravated BLM-induced lung injury and fibrosis. **A** The concentrations of L-ficolin and M-ficolin in plasma were measured by biochemical kits. *ILD vs. Healthy control, *P < 0.05, **P < 0.01, ***P < 0.001, ****P < 0.0001. **B** The Survival curves of mice after BLM induction. **C** The histopathological changes in the lung were observed by HE staining. **D** Pulmonary interstitial fibrosis was detected by Masson staining. **E**, **F** The concentrations of total protein in BALF and hydroxyproline in lung were determined by biochemical kits. **G** The expression level of α-SMA in lung was detected by Western blot. **H**, **I** The percentage of Fcn B positive cells in lung macrophages and neutrophils was detected by flow cytometry. The experiment was repeated three times (*n* = 10 mice/group). The data were presented as the mean ± SD. *WT+BLM vs. WT, *P < 0.05, **P < 0.01, ***P < 0.001, ****P < 0.0001; ^#^*Fcnb*^*-/-*^+BLM vs. WT+BLM, ^#^*P* < 0.05, ^##^*P* < 0.01, ^###^*P* < 0.001, ^####^*P* < 0.0001.

Firstly, our clinical data showed that there was no significant difference in the concentration of L-ficolin (homologous of Fcn A) in the plasma of interstitial lung disease (ILD) patients compared with healthy people, while that of M-ficolin (homologous of Fcn B) increased markedly (Fig. 1A). To explore the effect of Fcn B on lung injury, WT, *Fcna*^*-/-*^, and *Fcnb*^*-/-*^ mice were stimulated with BLM. Before BLM induction, the survival rates of WT, *Fcna*^*-/-*^, and *Fcnb*^*-/-*^ mice were all 100%. However, the survival rate of BLM-induced WT mice was significantly reduced, while that of BLM-induced *Fcnb*^*-/-*^ (rather than *Fcna*^*-/-*^) mice reduced slightly (Fig. 1B). HE staining proved that the alveolar structures were damaged to varying degrees, the alveolar wall was thickened, and accompanied by inflammatory infiltration after BLM induction, which indicated the successful construction of the lung injury model. Compared with WT+BLM group, the alveolar structure of *Fcnb*^*-/-*^+BLM group was relatively intact, with the least inflammatory infiltration (Fig. 1C). Masson staining representative of tissue fibrosis showed that only the lung interstitium of the *Fcnb*^*-/-*^+BLM group showed a smaller amount of collagen fiber deposition than that in the WT+BLM group (Fig. 1D). Similarly, the total protein concentrations in BALF and hydroxyproline concentration in lung tissues of *Fcnb*^*-/-*^+BLM group were significantly lower than that in the WT+BLM group (Fig. 1E, F). In addition, α-SMA expressions in the lung of *Fcnb*^*-/-*^+BLM group were significantly downregulated compared with the WT+BLM group (Fig. 1G). After BLM induction, increased Fcn B expressions were mainly derived from macrophages (partially from neutrophils) in the lung and blood of WT and *Fcna*^*-/-*^ mice (Fig. 1H, I and Supplementary Fig. S1A, B). The number of macrophages in the lung was increased after BLM induction, compared with the BLM-induced WT mice, the number of macrophages in the lung decreased obviously in BLM-induced *Fcnb*^*-/-*^ mice. However, neutrophils in the lungs of all groups were not significantly different (Supplementary Fig. S1C, D). These results proved that macrophage-derived Fcn B aggravated BLM-induced lung injury and fibrosis.

### Fcn B promoted autophagy and ferroptosis in BLM-induced lung injury model

**Fig. 2:**
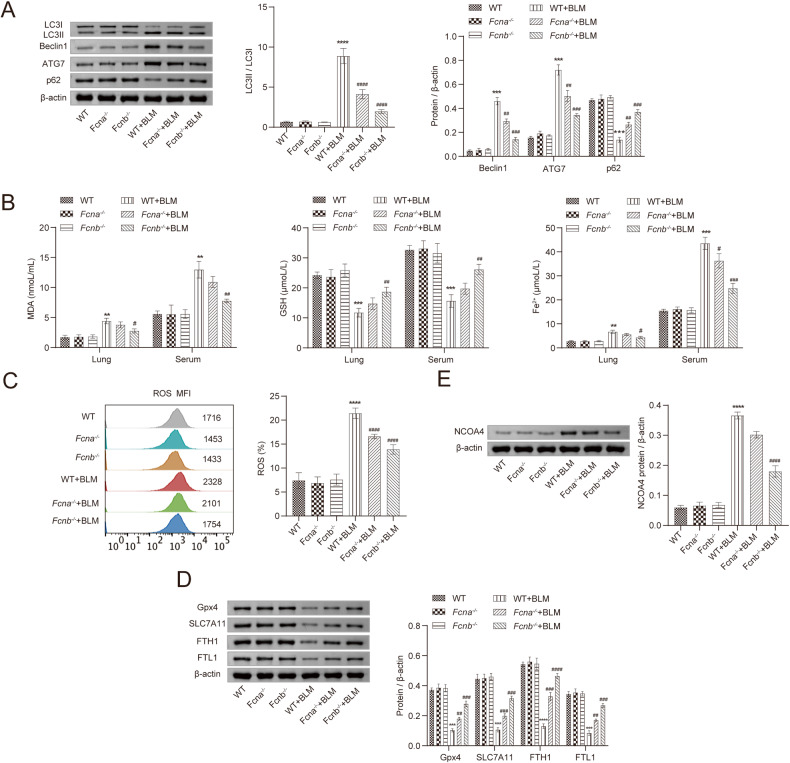
Fcn B promoted BLM-induced autophagy and ferroptosis in lung. **A** The expressions of LC3II/LC3I, Beclin1, ATG7, and p62 in lung were detected by Western blot. **B** The concentrations of MDA, GSH, and Fe^2+^ in lung and serum were detected by biochemical kits. **C** ROS levels in lung were determined by flow cytometry. **D**, **E** The expressions of GPX4, SLC7A11, FTH1, FTL1, and NCOA4 in lung were detected by Western blot. The experiment was repeated three times (*n* = 10 mice/group). The data were presented as the mean ± SD. *WT+BLM vs. WT, *P < 0.05, **P < 0.01, ***P < 0.001, ****P < 0.0001; ^#^*Fcnb*^*-/-*^+BLM vs. WT+BLM, ^#^*P* < 0.05, ^##^*P* < 0.01, ^###^*P* < 0.001, ^####^*P* < 0.0001.

Next, to explore the causes of Fcn B exacerbating lung injury, we examined the levels of markers associated with autophagy and ferroptosis in mice model. Compared with the WT+BLM group, the lung tissues of *Fcna*^*-/-*^+BLM and *Fcnb*^*-/-*^+BLM groups displayed reduced expressions of LC3II/LC3I, Beclin1, ATG7, and increased expression of p62 (Fig. 2A). While the concentrations of MDA and Fe^2+^ decreased, GSH concentrations increased significantly in the lung and serum of *Fcnb*^*-/-*^+BLM group alone compared with WT+BLM group (Fig. 2B). Then, the ROS levels of lung tissues were detected, which showed the *Fcnb*^*-/-*^+BLM group was significantly lower than the WT+BLM group (Fig. 2C). In addition, compared with the WT+BLM group, the lung tissues in *Fcnb*^*-/-*^+BLM group showed obviously increased GPX4, SLC7A11, FTH1, FTL1, and decreased NCOA4 expressions (Fig. 2D, E). Therefore, these results suggested that Fcn B could promote autophagy and ferroptosis in the BLM-induced lung injury model.

### BLM-induced AMs exosomes accelerated Fcn B expression, autophagy, and ferroptosis in lung epithelial cells

**Fig. 3:**
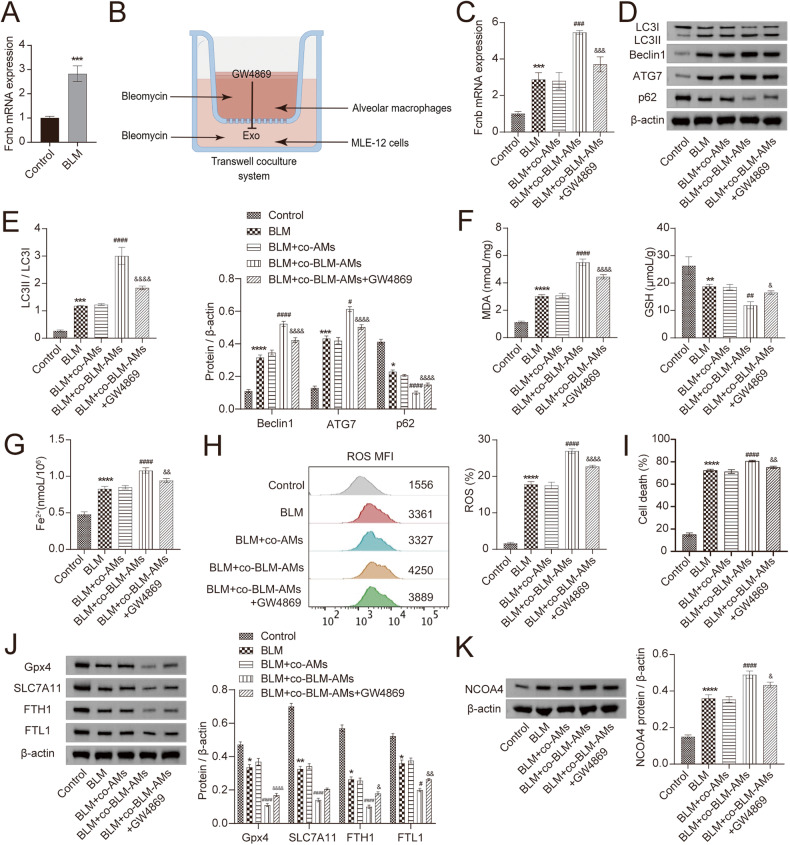
BLM-induced AMs exosomes increased Fcn B expression and promoted autophagy and ferroptosis in lung epithelial cells. **A** The mRNA expression of Fcn B in AMs was detected by RT-qPCR. **B** A sketch map of the Transwell coculture system. **C** The mRNA expression of Fcn B in lung epithelial cells was detected by RT-qPCR. **D**, **E** The expressions of LC3II/LC3I, Beclin1, ATG7, and p62 in lung epithelial cells were detected by Western blot. **F**, **G** The concentrations of MDA, GSH, and Fe^2+^ in lung epithelial cells were detected by biochemical kits. **H** ROS levels in lung epithelial cells were determined by flow cytometry. **I** The death rate of lung epithelial cells was determined by an LDH assay kit. **J**, **K** The expressions of GPX4, SLC7A11, FTH1, FTL1, and NCOA4 in lung epithelial cells were detected by Western blot. The experiment was repeated three times. The data were presented as the mean ± SD. *BLM vs. Control, *P < 0.05, **P < 0.01, ***P < 0.001, ****P < 0.0001; ^#^BLM+co-BLM-AMs vs. BLM+co-AMs, ^#^*P* < 0.05, ^##^*P* < 0.01, ^###^*P* < 0.001, ^####^*P* < 0.0001; & BLM+co-BLM-AMs+GW4869 vs. BLM+co-BLM-AMs, ^&^*P* < 0.05, ^&&^*P* < 0.01, ^&&&^*P* < 0.001, ^&&&&^*P* < 0.0001.

Lung epithelial cell death is proven to be the initial cause of lung injury and pulmonary fibrosis [41]. Therefore, we treated lung epithelial cells with BLM in vitro and detected the levels of autophagy and ferroptosis-related markers after co-culture with BLM-treated AMs. Moreover, exosome inhibitor GW4869 was applied to explore its effects on the functions of AMs exosomes. The mRNA expression of Fcn B in AMs was upregulated after BLM induction (Fig. 3A). In the Transwell coculture system, the addition of GW4869 inhibited the secretion and migration of exosomes from BLM-induced AMs in the upper chamber to BLM-induced lung epithelial cells in the lower chamber (Fig. 3B). When BLM-treated lung epithelial cells were co-cultured with BLM-treated AMs, the transcription of Fcn B was significantly increased. Unfortunately, this trend was reversed by the usage of GW4869 (Fig. 3C). Subsequently, Western blot results showed that the expressions of LC3II/LC3I, Beclin1, and ATG7 were upregulated, and p62 were downregulated after co-culture of BLM-treated lung epithelial cells with BLM-treated AMs. However, the addition of GW4869 reversed these changes (Fig. 3D, E). Biochemical detection further indicated that co-culture with BLM-treated AMs significantly increased the concentrations of MDA and Fe^2+^ in BLM-treated lung epithelial cells while decreasing that of GSH. However, the presence of GW4869 decreased the concentrations of MDA and Fe^2+^ but increased that of GSH (Fig. 3F, G). In addition, the ROS levels and death rate in BLM-treated lung epithelial cells also increased significantly after co-culture with BLM-treated AMs but decreased under the condition of GW4869 (Fig. 3H, I). Meanwhile, we found that after co-culture of BLM-treated lung epithelial cells with BLM-treated AMs, the expressions of GPX4, SLC7A11, FTH1, and FTL1 were significantly downregulated, while NCOA4 expression was significantly upregulated. However, the application of GW4869 conversed the above results (Fig. 3J, K). These results indicated that the regulation of Fcn B in AMs might act as a mechanism to participate in autophagy and ferroptosis, thereby alleviating BLM-induced lung epithelial cell injury.

### BLM-induced AMs mediated lung epithelial cells autophagy and ferroptosis through Fcn B transported by exosomes

**Fig. 4:**
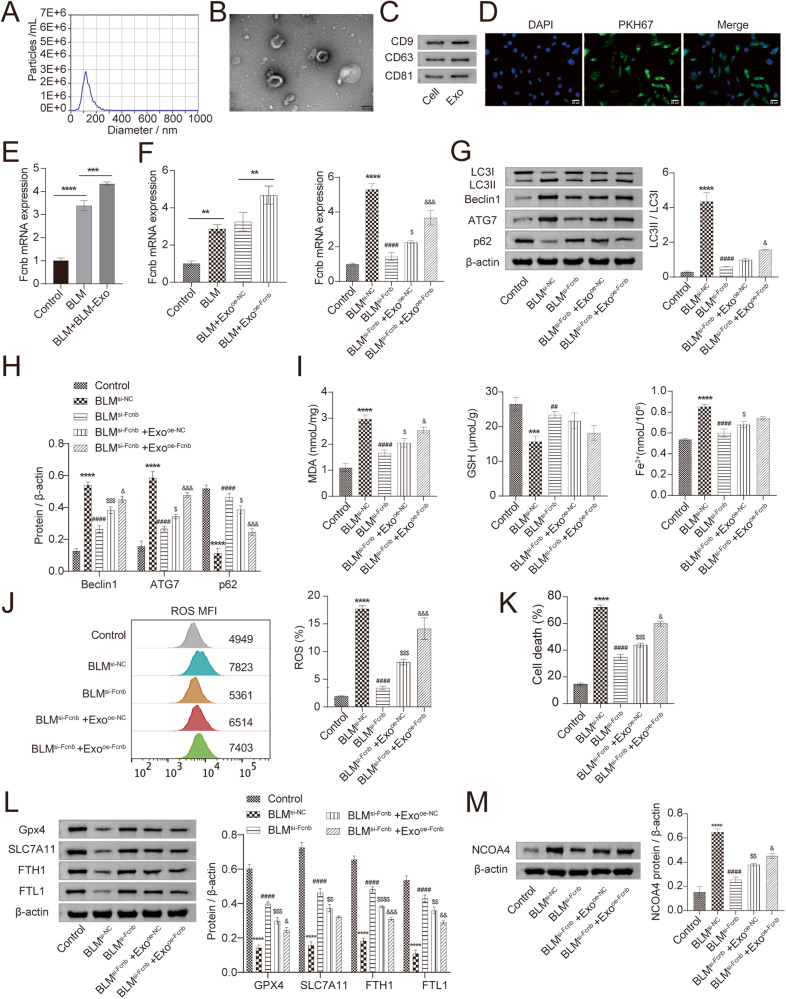
Fcn B transported by BLM-induced AMs exosomes contributed to lung epithelial cells autophagy and ferroptosis. **A** The particle size of exosomes was detected by NTA. **B** The morphology of exosomes was characterized by TEM. **C** The expressions of CD9, CD63, and CD81 in exosomes were characterized by Western blot. **D** Exosome uptake experiment. **E**, **F** The mRNA expression of Fcn B in lung epithelial cells was detected by RT-qPCR. **G**, **H** The expressions of LC3II/LC3I, Beclin1, ATG7, and p62 in lung epithelial cells were detected by Western blot. **I** The concentrations of MDA, GSH, and Fe^2+^ in lung epithelial cells were detected by biochemical kits. **J** ROS levels in lung epithelial cells were determined by flow cytometry. **K** The death rate of lung epithelial cells was determined by an LDH assay kit. **L**, **M** The expressions of GPX4, SLC7A11, FTH1, FTL1, and NCOA4 in lung epithelial cells were detected by Western blot. The experiment was repeated three times. The data were presented as the mean ± SD. *BLM^si-NC^ vs. Control, *P < 0.05, **P < 0.01, ***P < 0.001, ****P < 0.0001; ^#^BLM^si-Fcnb^ vs. BLM^si-NC^, ^#^*P* < 0.05, ^##^*P* < 0.01, ^###^*P* < 0.001, ^####^*P* < 0.0001; ^$^BLM^si-Fcnb^ +Exo^oe-NC^ vs. BLM^si-Fcnb^, ^$^*P* < 0.05, ^$$^*P* < 0.01, ^$$$^*P* < 0.001, ^$$$$^*P* < 0.0001; & BLM^si-Fcnb^ +Exo^oe-Fcnb^ vs. BLM^si-Fcnb^ +Exo^oe-NC^, ^&^*P* < 0.05, ^&&^*P* < 0.01, ^&&&^*P* < 0.001, ^&&&&^*P* < 0.0001.

Since Fcn B derived from pulmonary macrophages played an important role in BLM-induced mice model. Therefore, to investigate the connection between AMs and Fcn B, we extracted exosomes and carried out a series of characterizations. NTA results showed that most exosomes ranged from 40 to 160 nm in size (Fig. 4A). TEM images showed that exosomes were typically saucer-like with a single distribution (Fig. 4B). Western blot further showed that specific markers CD9, CD63, and CD81 were expressed in exosomes (Fig. 4C). Successful uptake of PKH67-labeled exosomes by lung epithelial cells and distribution around the nucleus were all observed (Fig. 4D). The mRNA expression of Fcn B was upregulated in BLM-treated lung epithelial cells after co-culture with BLM-treated AMs-derived exosomes (Fig. 4E). After co-culture of BLM-treated lung epithelial cells with oe-Fcnb-transfected AMs-derived exosomes, the mRNA expression of Fcn B was more significantly increased. After lung epithelial cells were transfected with si-Fcnb and treated with BLM, the mRNA expression of Fcn B was significantly inhibited. However, co-culture with oe-Fcnb-transfected and BLM-treated AMs-derived exosomes reversed this effect (Fig. 4F). Subsequently, Western blot results showed that the transfection of si-Fcnb could significantly downregulate the expressions of LC3II/LC3I, Beclin1, and ATG7 and upregulate that of p62 in BLM-treated lung epithelial cells. However, contrary results were obtained when co-cultured with oe-Fcnb-transfected and BLM-treated AMs-derived exosomes (Fig. 4G, H). In addition, biochemical detection results showed that the inhibitory effects of si-Fcnb on MDA and Fe^2+^ concentrations and the promotion of GSH in BLM-treated lung epithelial cells were reversed after co-culture with oe-Fcnb-transfected and BLM-treated AMs-derived exosomes (Fig. 4I). Similarly, the ROS level and death rate in si-Fcnb-transfected and BLM-treated lung epithelial cells were significantly increased after co-culture with oe-Fcnb-transfected and BLM-treated AMs-derived exosomes (Fig. 4J, K). Western blot results showed that si-Fcnb transfection significantly promoted the expressions of GPX4, SLC7A11, FTH1, and FTL1 and inhibited that of NCOA4 in BLM-treated lung epithelial cells. However, this trend was reversed when co-cultured with oe-Fcnb-transfected and BLM-treated AMs-derived exosomes (Fig. 4L, M). These results illustrated that BLM-induced AMs mediated lung epithelial cells autophagy and ferroptosis through Fcn B transported by exosomes.

### Fcn B from BLM-induced AMs exosomes promoted lung epithelial cells autophagy and ferroptosis through the cGAS-STING pathway

**Fig. 5:**
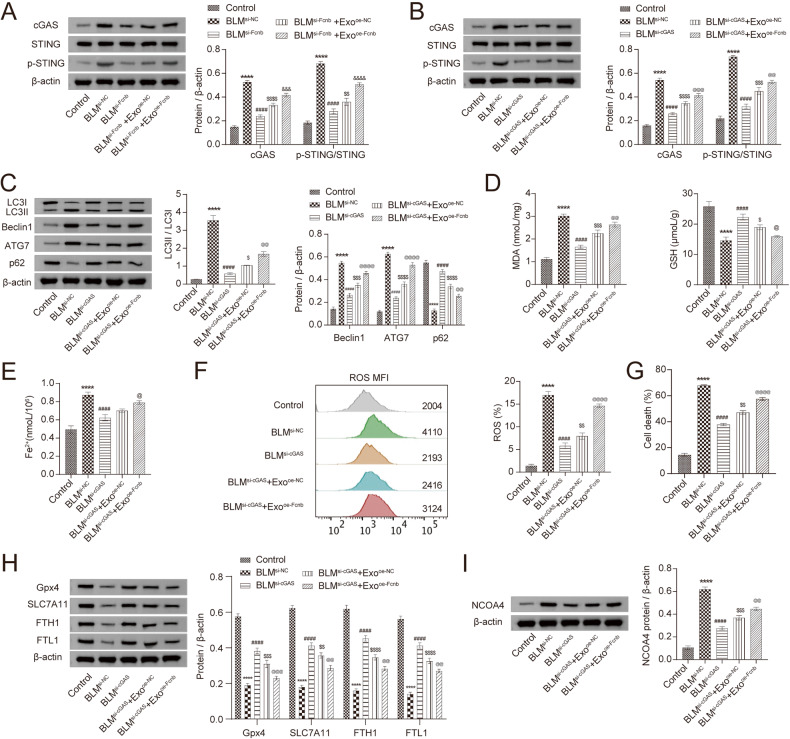
The cGAS-STING pathway mediated lung epithelial cells autophagy and ferroptosis that were promoted by Fcn B from BLM-induced AMs exosomes. **A**, **B** The expressions of cGAS, STING, and p-STING in lung epithelial cells were detected by Western blot. **C** The expressions of LC3I, LC3II, Beclin1, ATG7, and p62 in lung epithelial cells were detected by Western blot. **D**, **E** The concentrations of MDA, GSH, and Fe^2+^ in lung epithelial cells were detected by biochemical kits. **F** ROS levels in lung epithelial cells were determined by flow cytometry. **G** The death rate of lung epithelial cells was determined by an LDH assay kit. **H**, **I** The expression of GPX4, SLC7A11, FTH1, FTL1, and NCOA4 in lung epithelial cells was detected by Western blot. The experiment was repeated three times. The data were presented as the mean ± SD. *BLM^si-NC^ vs. Control, *P < 0.05, **P < 0.01, ***P < 0.001, ****P < 0.0001; ^#^BLM^si-cGAS^ vs. BLM^si-NC^, ^#^*P* < 0.05, ^##^*P* < 0.01, ^###^*P* < 0.001, ^####^*P* < 0.0001; ^$^BLM^si-cGAS^+Exo^oe-NC^ vs. BLM^si-cGAS^, ^*$*^*P* < 0.05, ^*$$*^*P* < 0.01, ^*$$$*^*P* < 0.001, ^*$$$$*^*P* < 0.0001; @ BLM^si-cGAS^+Exo^oe-Fcnb^ vs. BLM^si-cGAS^*+*Exo^oe-NC^, ^@^*P* < 0.05, ^@^^*@*^*P* < 0.01, ^@@@^*P* < 0.001, ^*@@*@@^*P* < 0.0001.

To clarify the potential mechanism of FcnB promoting autophagy and ferroptosis of lung epithelial cells, we intervened in the cGAS-STING pathway and analyzed the expression of pathway-related proteins. When si-Fcnb or si-cGAS-transfected lung epithelial cells were induced by BLM, the expressions of cGAS, LC3II/LC3I, Beclin1, and ATG7 were downregulated, and the phosphorylation of STING was also reduced, while the expression of p62 was upregulated. However, when they were co-cultured with oe-Fcnb-transfected and BLM-induced AMs, the expressions of cGAS, LC3II/LC3I, Beclin1, and ATG7 were upregulated, and the phosphorylation of STING was also increased, while the expression of p62 was downregulated (Fig. 5A–C). Further research found that the levels of MDA and Fe^2+^ were decreased while that of GSH increased in si-Fcnb or si-cGAS-transfected lung epithelial cells after BLM induction. However, after co-culture of oe-Fcnb-transfected and BLM-induced AMs, the levels of MDA and Fe^2+^ were increased, whereas that of GSH was the opposite (Fig. 5D, E). Similarly, when lung epithelial cells transfected with si-Fcnb or si-cGAS were induced by BLM, the level of ROS and cell death rate were both decreased. Contrarily, when they were co-cultured with oe-Fcnb-transfected and BLM-induced AMs, the level of ROS and cell death rate were both increased (Fig. 5F, G). The detection of ferroptosis-related proteins found that when the lung epithelial cells transfected with si-Fcnb or si-cGAS were induced by BLM, the expressions of GPX4, SLC7A11, FTH1, and FTL1 were upregulated, and that of NCOA4 was downregulated. Unfortunately, when they were co-cultured with oe-Fcnb-transfected and BLM-induced AMs, the expressions of GPX4, SLC7A11, FTH1, and FTL1 were downregulated, while that of NCOA4 was upregulated (Fig. 5H, I). These results indicated that the cGAS-STING pathway mediated lung epithelial cells autophagy and ferroptosis that were promoted by Fcn B from BLM-induced AMs exosomes.

### Lung epithelial cells ferroptosis promoted by Fcn B from BLM-induced AMs exosomes through the cGAS-STING pathway were autophagy-dependent

**Fig. 6:**
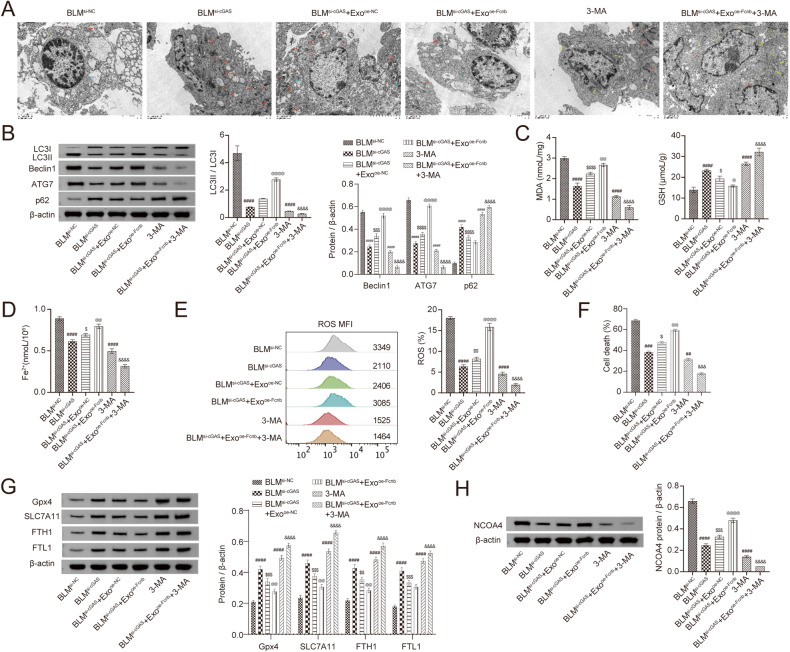
Inhibition of autophagy alleviated lung epithelial cells ferroptosis promoted by Fcn B from BLM-induced AMs exosomes through the cGAS-STING pathway. **A** Mitochondrial damage and autophagosomes were detected by TEM (The yellow arrow represented normal mitochondria, the red arrow represented damaged mitochondria, and the blue arrow represented autophagosomes). **B** The expressions of LC3II/LC3I, Beclin1, ATG7, and p62 in lung epithelial cells were detected by Western blot. **C**, **D** The concentrations of MDA, GSH, and Fe^2+^ in lung epithelial cells were detected by biochemical kits. **E** ROS levels in lung epithelial cells were determined by flow cytometry. **F** The death rate of lung epithelial cells was determined by an LDH assay kit. **G**, **H** The expressions of GPX4, SLC7A11, FTH1, FTL1, and NCOA4 in lung epithelial cells were detected by Western blot. The experiment was repeated three times. The data were presented as the mean ± SD. ^#^BLM^si-cGAS^ or 3-MA vs. BLM^si-NC^, ^#^*P* < 0.05, ^##^*P* < 0.01, ^###^*P* < 0.001, ^####^*P* < 0.0001; ^$^BLM^si-cGAS^+Exo^oe-NC^ vs. BLM^si-cGAS^, ^*$*^*P* < 0.05, ^*$$*^*P* < 0.01, ^*$$$*^*P* < 0.001, ^*$$$$*^*P* < 0.0001; ^@^BLM^si-cGAS^+Exo^oe-Fcnb^ vs. BLM^si-cGAS^+Exo^oe-NC^, ^@^*P* < 0.05, ^@@^*P* < 0.01, ^@@@^*P* < 0.001, ^@@@@^*P* < 0.0001; & BLM^si-cGAS^+Exo^oe-Fcnb^+3-MA vs. BLM^si-cGAS^+Exo^oe-Fcnb^, ^&^*P* < 0.05, ^&&^*P* < 0.01, ^&&&^*P* < 0.001, ^@@@@^*P* < 0.0001.

To further elucidate the relation of lung epithelial cells ferroptosis and autophagy, si-cGAS-transfected and BLM-induced lung epithelial cells were co-cultured with exosomes from different AMs and combined with autophagy inhibitor 3-MA for the study. Mitochondrial damage was reduced in si-cGAS-transfected and BLM-induced lung epithelial cells. When they were co-cultured with oe-Fcnb-transfected and BLM-treated AMs exosomes, mitochondrial damage was aggravated. However, the use of 3-MA reversed the damage caused by oe-Fcnb transfection to the cells, and most mitochondria in the cells remained in a normal state (Fig. 6A). Moreover, 3-MA reduced the autophagy promotion of Fcn B from BLM-induced AMs exosomes on si-cGAS-transfected and BLM-induced lung epithelial cells, exhibiting reduced LC3II/LC3I, Beclin1, and ATG7 expressions, and increased p62 expression (Fig. 6B). Next, oe-Fcnb-transfected and BLM-treated AMs exosomes promoted the accumulation of MDA and Fe^2+^ in si-cGAS-transfected and BLM-treated lung epithelial cells and inhibited the synthesis of GSH, whereas the addition of 3-MA showed the opposite effect (Fig. 6C, D). Similarly, the ROS level and death rate of si-cGAS-transfected and BLM-treated lung epithelial cells were both increased by oe-Fcnb-transfected and BLM-treated AMs exosomes, but this trend was changed by the addition of 3-MA (Fig. 6E, F). Additionally, the expressions of GPX4, SLC7A11, FTH1, and FTL1 were downregulated and that of NCOA4 was upregulated after si-cGAS-transfected and BLM-treated lung epithelial cells co-cultured with oe-Fcnb-transfected and BLM-treated AMs exosomes, but these changes conversed under the condition of 3-MA (Fig. 6G, H). These results displayed that inhibition of autophagy alleviated lung epithelial cells ferroptosis promoted by Fcn B from BLM-induced AMs exosomes through the cGAS-STING pathway.

### Fcn B transported from AMs exosomes facilitated lung injury and fibrosis *via* ferroptosis in BLM-induced mice model

**Fig. 7:**
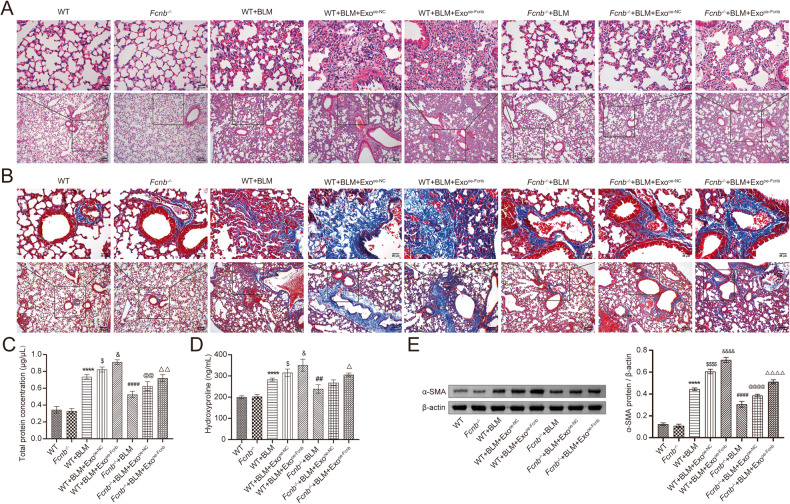
Fcn B transported from AMs exosomes facilitated lung injury and fibrosis in BLM-induced mice model. **A** The histopathological changes in the lung were observed by HE staining. **B** Pulmonary interstitial fibrosis was detected by Masson staining. **C**, **D** The concentrations of total protein in BALF and hydroxyproline in the lung were determined by biochemical kits. **E** The expression of α-SMA in lung was detected by Western blot. The experiment was repeated three times (*n* = 10 mice/group). The data were presented as the mean ± SD. * WT+BLM vs. WT, *P < 0.05, **P < 0.01, ***P < 0.001, ****P < 0.0001; ^$^WT+BLM+Exo^oe-NC^ vs. WT+BLM, ^$^*P* < 0.05, ^$$^*P* < 0.01, ^$$$^*P* < 0.001, ^$$$$^*P* < 0.0001; & WT+BLM+Exo^oe-Fcnb^ vs. WT+BLM+Exo^oe-NC^, ^&^*P* < 0.05, ^&&^*P* < 0.01, ^&&&^*P* < 0.001, ^&&&&^*P* < 0.0001; ^#^*Fcnb*^*-/-*^+BLM vs. WT+BLM, ^#^*P* < 0.05, ^##^*P* < 0.01, ^###^*P* < 0.001, ^####^*P* < 0.0001; @ *Fcnb*^*-/-*^+BLM+Exo^oe-NC^ vs. *Fcnb*^*-/-*^+BLM, ^@^*P* < 0.05, ^@@^*P* < 0.01, ^*@@*@^*P* < 0.001, ^@@@@^*P* < 0.0001; Δ *Fcnb*^*-/-*^+BLM+Exo^oe-Fcnb^ vs. *Fcnb*^*-/-*^+BLM+Exo^oe-NC^, ^Δ^*P* < 0.05, ^ΔΔ^*P* < 0.01, ^ΔΔΔ^*P* < 0.001, ^ΔΔΔΔ^*P* < 0.0001.

**Fig. 8:**
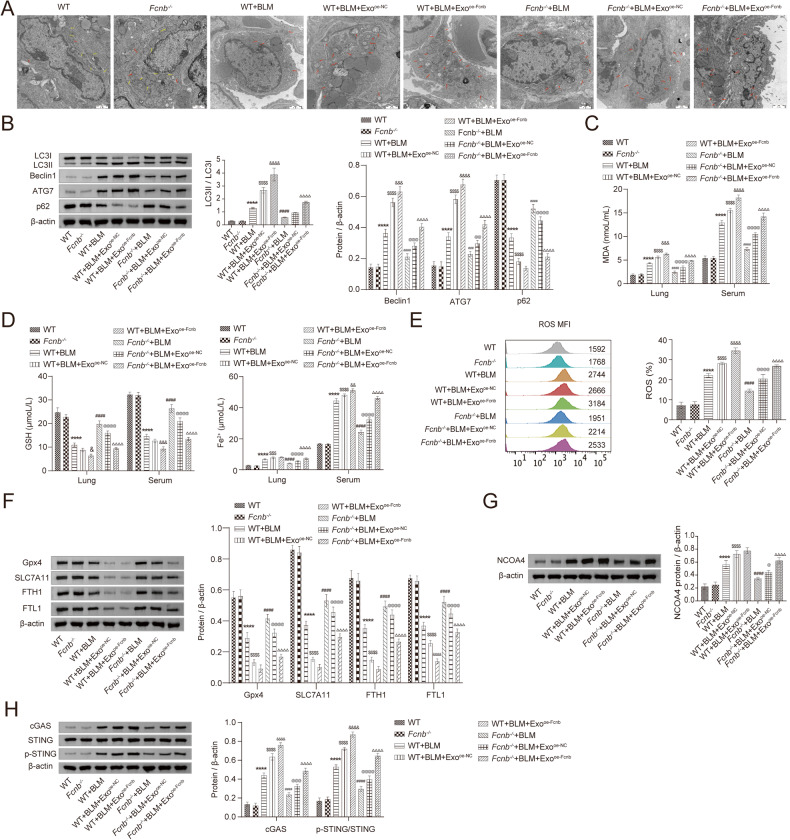
Fcn B transported from AMs exosomes facilitated ferroptosis in BLM-induced mice. **A** Mitochondrial damage and autophagosomes were detected by TEM (The red arrow represented damaged mitochondria). **B** The expressions of LC3II/LC3I, Beclin1, ATG7, and p62 in lung were detected by Western blot. **C**, **D** The concentrations of MDA, GSH, and Fe^2+^ in lung and serum were detected by biochemical kits. **E** ROS levels in lung were determined by flow cytometry. **F**, **G** The expression of GPX4, SLC7A11, FTH1, FTL1, and NCOA4 in lung were detected by Western blot. **H** The expressions of cGAS, STING, and p-STING in lung were detected by Western blot. The experiment was repeated three times (*n* = 10 mice/group). The data were presented as the mean ± SD. *WT+BLM vs. WT, *P < 0.05, **P < 0.01, ***P < 0.001, ****P < 0.0001; ^$^WT+BLM+Exo^oe-NC^ vs. WT+BLM, ^$^*P* < 0.05, ^$$^*P* < 0.01, ^$$$^*P* < 0.001, ^$$$$^*P* < 0.0001; & WT+BLM+Exo^oe-Fcnb^ vs. WT+BLM+Exo^oe-NC^, ^&^*P* < 0.05, ^&&^*P* < 0.01, ^&&&^*P* < 0.001, ^&&&&^*P* < 0.0001; ^#^*Fcnb*^*-/-*^+BLM vs. WT+BLM, ^#^*P* < 0.05, ^##^*P* < 0.01, ^###^*P* < 0.001, ^####^*P* < 0.0001; @ *Fcnb*^*-/-*^+BLM+Exo^oe-NC^ vs. *Fcnb*^*-/-*^+BLM, ^@^*P* < 0.05, ^@@^*P* < 0.01, ^*@@*@^*P* < 0.001, ^@@@@^*P* < 0.0001; ^Δ^*Fcnb*^*-/-*^+BLM+Exo^oe-Fcnb^ vs. *Fcnb*^*-/-*^+BLM+Exo^oe-NC^, ^Δ^*P* < 0.05, ^ΔΔ^*P* < 0.01, ^ΔΔΔ^*P* < 0.001, ^ΔΔΔΔ^*P* < 0.0001.

To investigate the function of Fcn B derived from exosomes of AMs in vivo, WT and *Fcnb*^*-/-*^ mice were stimulated by BLM and then injected with oe-Fcnb-transfected and BLM-induced AMs exosomes into the tail vein for intervention. HE staining showed the alveolar structure in the *Fcnb*^*-/-*^+BLM group was more integrated with less inflammatory infiltration than that in the WT+BLM group. However, compared with the *Fcnb*^*-/-*^+BLM+Exo^oe-NC^ group, the alveolar structure in the *Fcnb*^*-/-*^+BLM+Exo^oe-Fcnb^ group was incomplete and inflammatory infiltration increased (Fig. 7A). Masson staining also revealed that increased pulmonary interstitial fibrosis was observed in the *Fcnb*^*-/-*^+BLM+Exo^oe-Fcnb^ group (Fig. 7B). Additionally, we found increased total protein concentration of BALF, increased hydroxyproline and α-SMA expressions of lung tissues in the *Fcnb*^*-/-*^+BLM+Exo^oe-Fcnb^ group than that in the *Fcnb*^*-/-*^+BLM+Exo^oe-NC^ group (Fig. 7C–E). Additionally, the number of damaged mitochondria increased lung tissues in the *Fcnb*^*-/-*^+BLM+Exo^oe-Fcnb^ group than that in the *Fcnb*^*-/-*^+BLM+Exo^oe-NC^ group (Fig. 8A). Further, the expressions of LC3II/LC3I, Beclin1, and ATG7 were significantly upregulated, and that of p62 downregulated in the lung of *Fcnb*^*-/-*^+BLM+Exo^oe-Fcnb^ group compared with the *Fcnb*^*-/-*^+BLM+Exo^oe-NC^ group (Fig. 8B). Biochemical detection showed that compared with the *Fcnb*^*-/-*^+BLM+Exo^oe-NC^ group, concentrations of MDA and Fe^2+^ in lung tissues and serum in the *Fcnb*^*-/-*^+BLM+Exo^oe-Fcnb^ group were significantly increased, while that of GSH was decreased (Fig. 8C, D). Flow cytometry displayed that the ROS level in lung tissues in the *Fcnb*^*-/-*^+BLM+Exo^oe-Fcnb^ group was higher than that in the *Fcnb*^*-/-*^+BLM+Exo^oe-NC^ group (Fig. 8E). Besides, compared with that in the *Fcnb*^*-/-*^+BLM+Exo^oe-NC^ group, the expressions of GPX4, SLC7A11, FTH1, and FTL1 in lung tissues of the *Fcnb*^*-/-*^+BLM+Exo^oe-Fcnb^ group were significantly downregulated, while that of NCOA4 was upregulated (Fig. 8F, G). Moreover, the expression of cGAS and the phosphorylation of STING were remarkedly higher in the *Fcnb*^*-/-*^+BLM+Exo^oe-Fcnb^ group than those in the *Fcnb*^*-/-*^+BLM+Exo^oe-NC^ group (Fig. 8H). Together, these results showed that Fcn B transported from AMs exosomes facilitated lung injury and fibrosis through ferroptosis in BLM-induced mice model.

## Discussion

Lung injury, due to its complex pathogenesis, and lack of effective treatment, is a major problem in pulmonary diseases that needs to be solved urgently. Our previous results displayed an obvious role of Fcn A in acute lung injury [26, 27]. However, the effect and mechanism of Fcn B on lung injury remain unclear. Our clinical data first found an increase of M-ficolin (rather than L-ficolin) in plasma in ILD patients indicating the role of Fcn B on lung inflammation. The stimulant used in the model is BLM, as a common inducer to construct lung injury models, which could also cause cell necrosis and pulmonary fibrosis [42, 43]. Diffuse alveolar cell injury and inflammatory infiltration are the main pathological features of lung injury [44]. LPS-treated mice showed changes in the lung, such as edema, hemorrhage, and alveolar collapse [45]. After BLM induction, the alveolar structure of *Fcnb*^*-/-*^ mice was relatively complete, with less inflammatory infiltration and a small amount of collagen fiber deposition, indicating that Fcn B could aggravate BLM-induced lung tissue lesions. BLM-induced activated myofibroblasts are the most important effector cells in pulmonary fibrosis, capable of producing extracellular matrix (ECM) to induce collagen fiber deposition [46]. Total protein in BALF is one of the markers of the destruction of the alveolar-capillary barrier, which can reflect the degree of lung tissue damage [47]. Hydroxyproline is a unique amino acid in collagen fibers, which indirectly reflects the deposition level of collagen fibers [48]. The abnormally high expression of α-SMA is a marker of fibroblast differentiation into myofibroblasts [49]. Therefore, after BLM induction, the concentrations of total protein in BALF, hydroxyproline, and α-SMA expressions in lung tissues were decreased in *Fcnb*^*-/-*^ mice, verifying that Fcn B could aggravate BLM-induced pulmonary fibrosis. Studies have shown that macrophages and neutrophils are the main sources of Fcn A and Fcn B, and Fcn B are also partially expressed in lung epithelial cells [26, 50]. Consequently, macrophages and neutrophils were selected in this study to detect the expression of Fcn B. Flow cytometry showed that Fcn B was highly expressed in macrophages of BLM-induced WT and *Fcna*^*-/-*^ mice, especially in lung macrophages. Besides, the number of lung macrophages was significantly decreased in the *Fcnb*^*-/-*^+BLM group. These results indicated that in this model, secretory Fcn B is mainly derived from lung macrophage and affects the number of macrophages in return.

Ferroptosis is a type of programmed cell death that depends on iron and ROS. Many studies have confirmed that ferroptosis is closely related to the occurrence of lung injury and autophagy [14, 51, 52]. Autophagy is a lysosomal-mediated intracellular protein degradation process involving many autophagy-related factors and signaling pathways. Among them, the transformation of LC3I to LC3II is a key indicator for judging the level of autophagy activity of cells and the late formation of autophagosomes [53]. Beclin1 is a key protein in the early formation of autophagosomes and an important target in the regulation of autophagy [54]. ATG7 is a ubiquitin ligase in the autophagy process evaluating the autophagy level [55]. p62 is a receptor protein, and its expression level is inversely proportional to autophagy activity [56]. In this study, compared with that in the WT+BLM group, the expressions of LC3II/LC3I, Beclin1, and ATG7 of lung tissues in the *Fcnb*^*-/-*^+BLM group were downregulated, while p62 expression was upregulated, indicating that Fcn B could promote BLM-induced autophagy. Studies have shown that ferritinophagy is a process that degrades ferritin, such as FTL1 and FTH1, with NCOA4 as cargo receptor protein, and then releases free iron [18]. When intracellular iron metabolism is unbalanced, Fe^2+^ produces ROS through the Fenton reaction, which directly combines with polyunsaturated fatty acids to lead to the large accumulation of lipid peroxides (LPO) and induce ferroptosis [57]. LPO is eventually metabolized into MDA, which leads to cell death [58]. Depletion or decreased GSH and GPX4 activity in cells can lead to a declined ability to clear LPO, thus inducing ferroptosis [59]. In addition, the downregulation of SLC7A11 expression was found to lead to ferroptosis [60]. Here, compared with those in the WT+BLM group, ROS, MDA, Fe^2+^, and NCOA4 expressions in the *Fcnb*^*-/-*^+BLM group decreased, while GSH, GPX4, SLC7A11, FTH1, and FTL1 increased. These results indicated that in the BLM-induced lung injury model, Fcn B promoted ferroptosis mediated by two pathways.

Damaged lung epithelial cells and activation of AMs are thought to be the initiating factor of lung injury [61]. Our study showed that BLM-induced AMs promoted ferroptosis in BLM-treated lung epithelial cells. Recent studies have shown that exosomes derived from AMs of BALF can promote pneumonia and lung injury [62]. Additionally, exosomes obtained after LPS stimulation of AMs can lead to increased total protein in BALF and inflammatory infiltration in lung [63]. In this study, AMs-derived exosomes were typically saucer-like with a size from 40 to 160 nm, which could express specific markers CD9, CD63, and CD81 and successfully loaded with Fcn B. The transfection of si-Fcnb inhibited the occurrence of autophagy and ferroptosis in BLM-induced lung epithelial cells, but this effect was reversed by oe-Fcnb-transfected and BLM-treated AMs-derived exosomes, suggesting that BLM-induced AMs mediated lung epithelial cells ferroptosis through Fcn B transported by exosomes. Studies have shown that the cGAS-STING pathway plays an important regulatory role in the regulation of autophagy, ferritinophagy, ferroptosis, and inflammation [64–67]. In addition, studies on alleviating lung injury by inhibiting the activation of the cGAS-STING pathway have been reported, but the literature is relatively few [35–37, 68]. In this study, by silencing cGAS, it was found that the activation of the cGAS-STING pathway in BLM-induced lung epithelial cells was inhibited, and the levels of autophagy and ferroptosis were also decreased. However, Fcn B transported by exosomes from BLM-induced AMs reversed the trend. These results proved that the cGAS-STING pathway mediated lung epithelial cells autophagy and ferroptosis that were promoted by Fcn B from BLM-induced AMs exosomes. In some lung injury models, the prophylactic use of the autophagy inhibitor 3-MA alleviated excessive inflammatory injury by blocking the autophagy response [69, 70]. So, to determine the effect of autophagy on Fcn B function and ferroptosis, we treated si-cGAS-transfected and BLM-induced lung epithelial cells with the autophagy inhibitor 3-MA and oe-Fcnb-transfected and BLM-treated AMs exosomes. The results showed a significant decrease in ferroptosis activity, suggesting autophagy-dependent ferroptosis in lung epithelial cells was mediated by Fcn B through the activation of the cGAS-STING pathway. Further, BLM-induced mice were injected with exosomes from oe-Fcnb-transfected and BLM-induced AMs *via* tail vein to verify the role of Fcn B in vivo. The results showed more severe lung injury, increased inflammatory infiltration, and increased levels of autophagy and ferroptosis, suggesting that AMs-derived exosomes facilitated lung injury, fibrosis, and ferroptosis by transporting Fcn B in vivo.

**Fig. 9:**
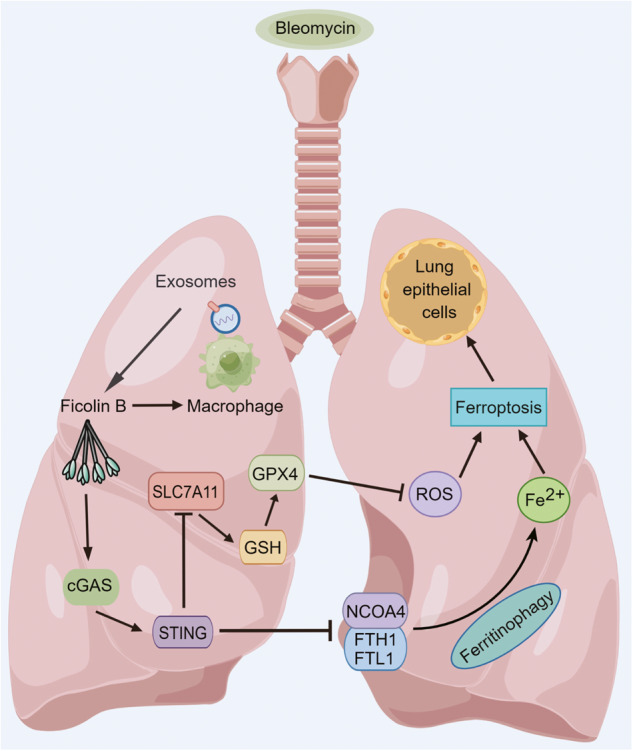
Fcn B transported by exosomes from AMs exacerbated BLM-induced lung injury by damaging lung epithelial cells in a ferroptosis manner through the cGAS-STING signaling pathway.

In summary, this is the first report investigating the role of Fcn B in BLM-induced lung injury, which showed that Fcn B transported by exosomes from AMs could exacerbate BLM-induced lung injury by promoting lung epithelial cells ferroptosis through the cGAS-STING pathway (Fig. 9).

Although this study has provided important findings on the role of Fcn B in promoting ferroptosis in BLM-induced lung injury, there are several limitations to consider. One limitation of this study is that it only focuses on the involvement of autophagy and ferroptosis in BLM-induced lung injury, with limited attention to other forms of cell death. In BLM-induced lung injury, other forms of cell death, such as apoptosis and pyroptosis, also play important roles. BLM can promote cell apoptosis and pyroptosis in lung cells through multiple pathways and processes [71–80]. Therefore, it will be interesting to investigate the regulation of cell apoptosis and pyroptosis by Fcn B in BLM-induced lung injury. Another limitation is that our study is centered around the cGAS-STING pathway as a potential mediator of Fcn B-regulated autophagy and ferroptosis in BLM-induced lung injury. However, the pathogenesis of lung injury is complex and multifactorial, and there are many other pathways that have not been explored in our study. Investigating the interactions between Fcn B, these pathways, and multiple modes of cell death can provide a more comprehensive understanding of the mechanisms underlying lung injury. Therefore, future studies should address these limitations to further elucidate the mechanisms of lung injury and the potential therapeutic implications of Fcn B.

## Conclusion

To conclude, our findings suggested that Fcn B exacerbated BLM-induced lung injury through the cGAS-STING pathway. This study will enrich the understanding of the role of Fcn B in lung injury, provide clues to the molecular mechanism of the occurrence and development of BLM-induced lung injury and provide new targets for its clinical treatment.

## Supplementary information

Supplementary information

Original Data File

Reproducibility checklist

## Data Availability

The data used to support the findings of this study are available from the corresponding author upon request.
