# Supplementary material for: Ficolin B secreted by alveolar macrophage exosomes exacerbates bleomycin-induced lung injury via ferroptosis through the cGAS-STING signaling pathway
Source: Cell Death Dis. 2023 Aug 30;14(8):577. doi: 10.1038/s41419-023-06104-4 (PMC10468535; doi:10.1038/s41419-023-06104-4)
Supplement: Supplementary file 1 — Supplementary information [file 41419_2023_6104_MOESM1_ESM.docx]

**Supplementary Information**

**
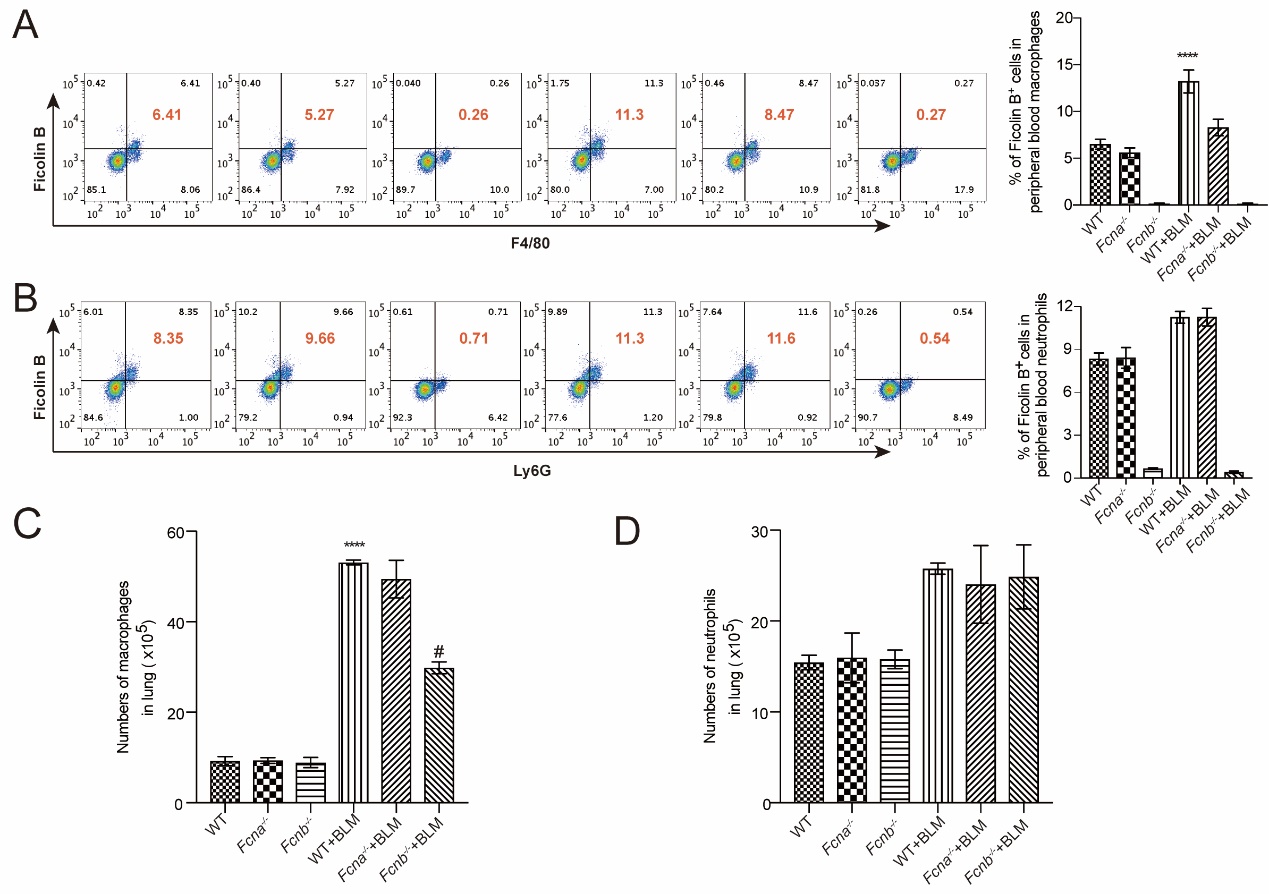
**

**Figures S1. Fcn B expression in blood and macrophage/neutrophil numbers in lung induced by BLM.** (A-B) The percentage of Fcn B positive cells in blood macrophages and neutrophils; (C-D) The number of macrophages and neutrophils in the lung. The experiment was repeated three times (n=10 mice/group). The data were presented as the mean±SD. * WT+BLM vs. WT, * *P*<0.05, ** *P*<0.01, *** *P*<0.001, **** *P*<0.0001; # *Fcnb^-/-^*+BLM vs. WT+BLM, # *P*<0.05, ## *P*<0.01, ### *P*<0.001, #### *P*<0.0001.
