# Supplementary material for: Ficolin B secreted by alveolar macrophage exosomes exacerbates bleomycin-induced lung injury via ferroptosis through the cGAS-STING signaling pathway
Source: Cell Death Dis. 2023 Aug 30;14(8):577. doi: 10.1038/s41419-023-06104-4 (PMC10468535; doi:10.1038/s41419-023-06104-4)

Figure 1G

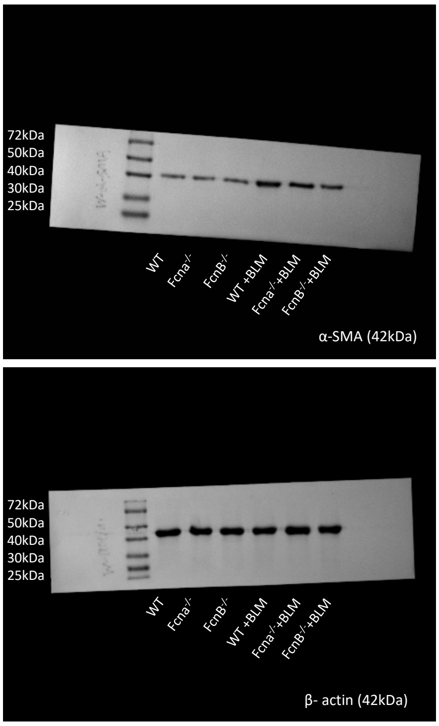

Figure 2A

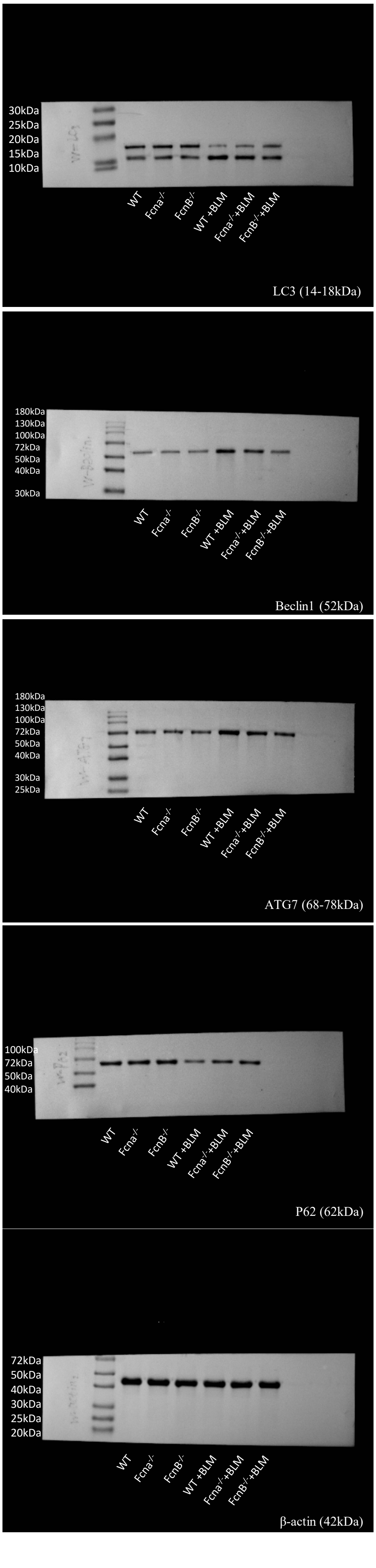

Figure 2E

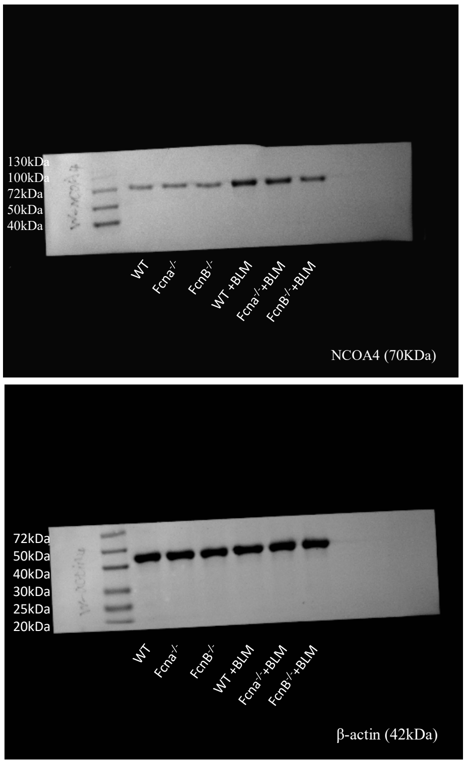

Figure 2D

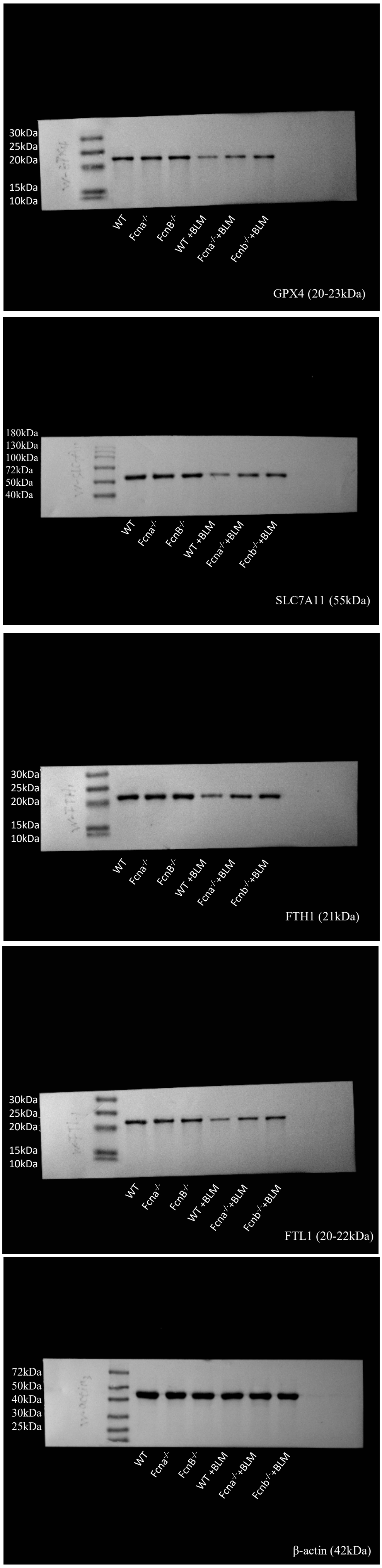

Figure 3D

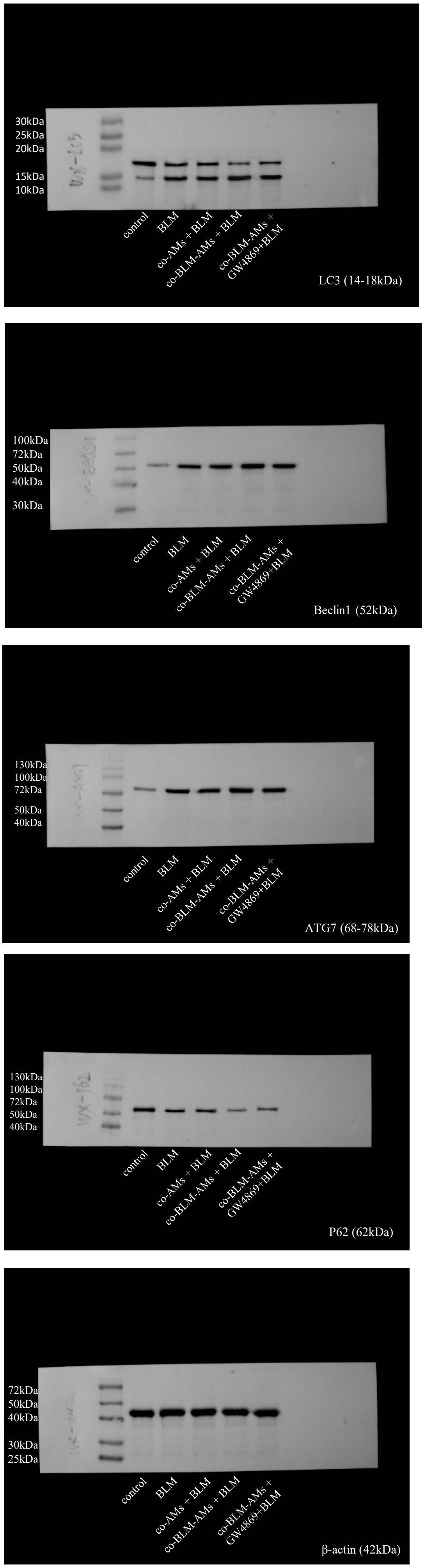

Figure 3J

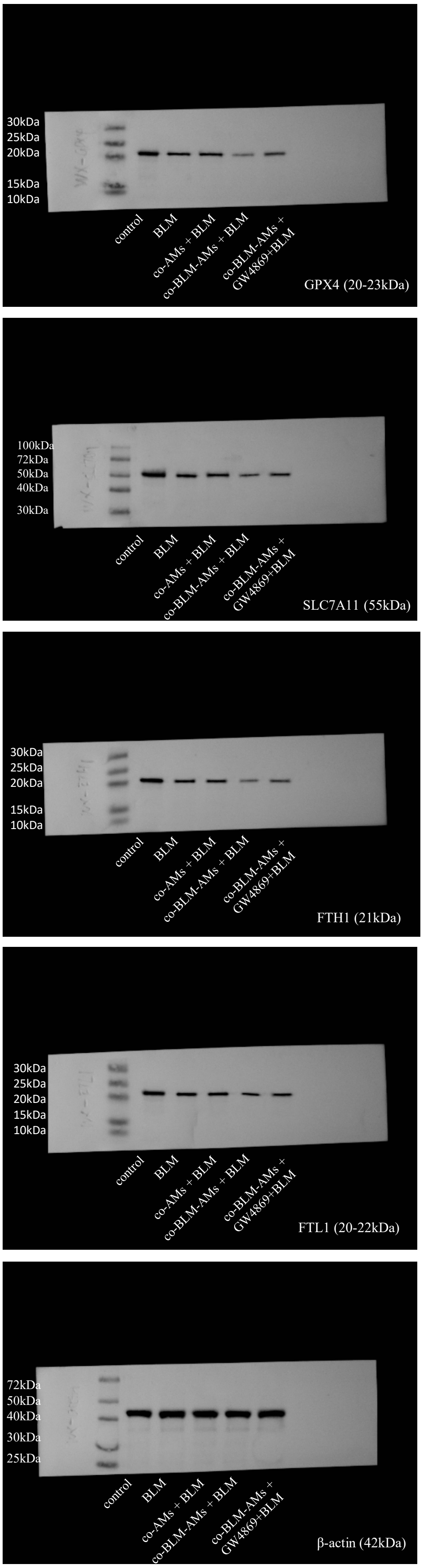

Figure 3K

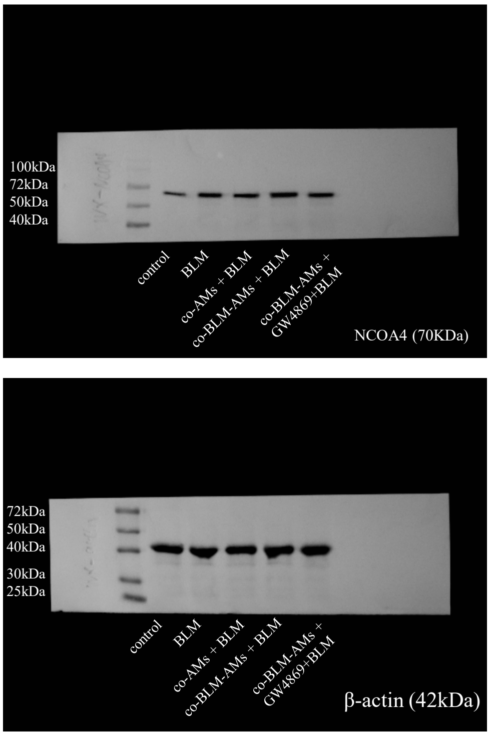

Figure 4C

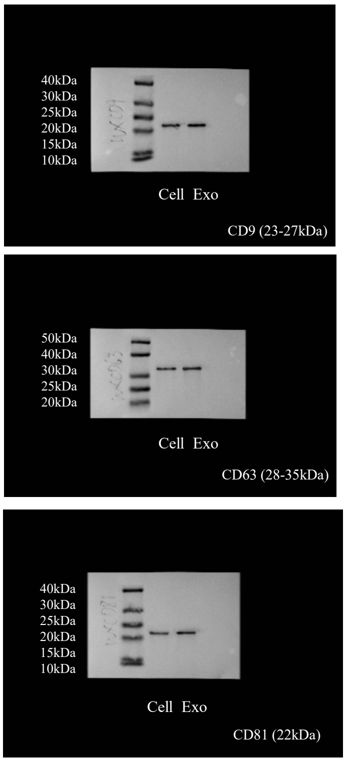

Figure 4G

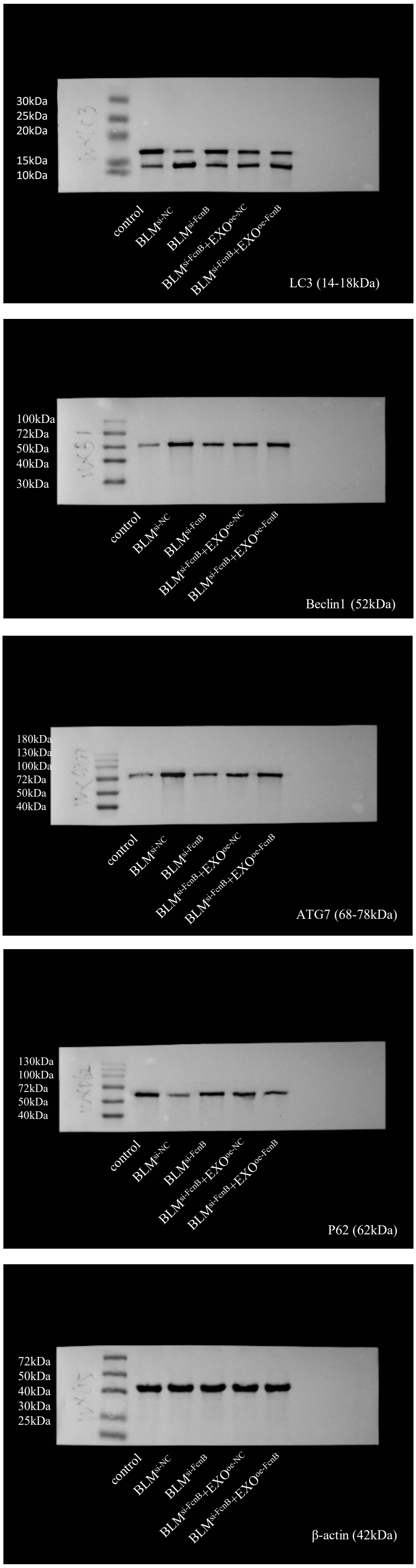

Figure 4L

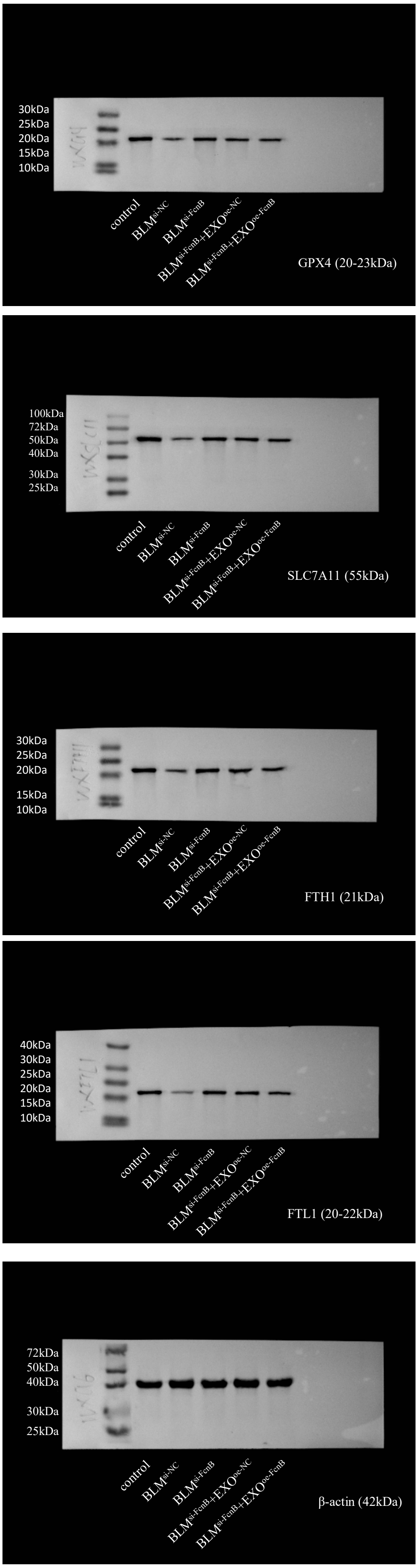

Figure 4M

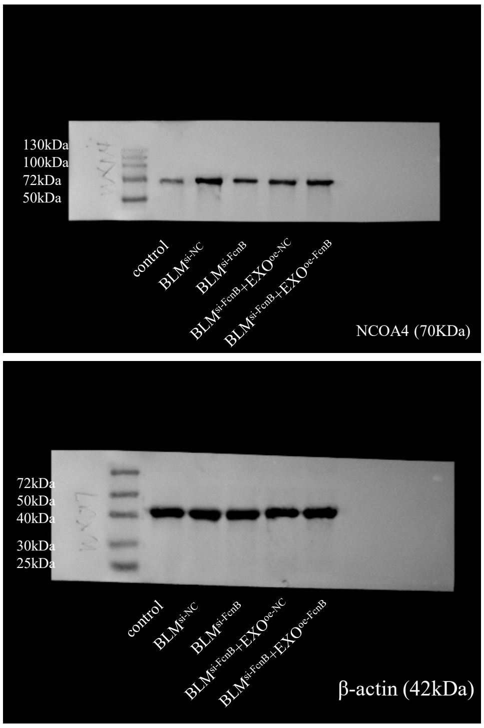

Figure 5A

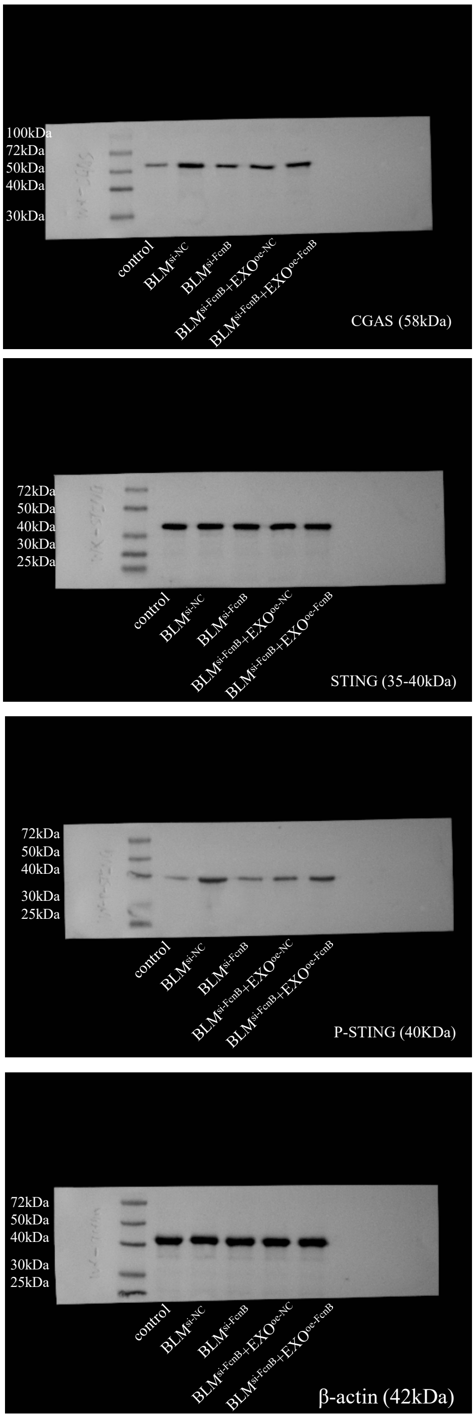

Figure 5B

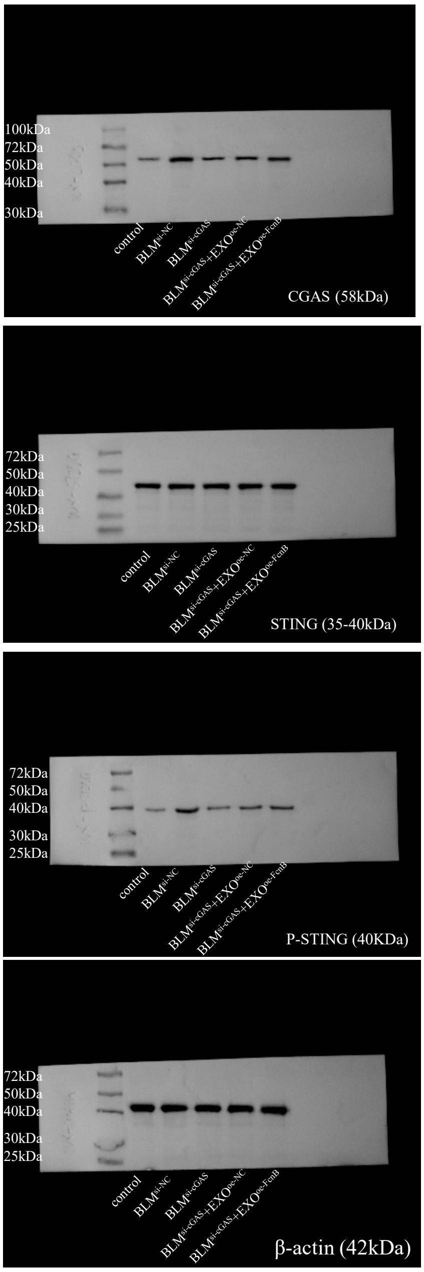

Figure 5C

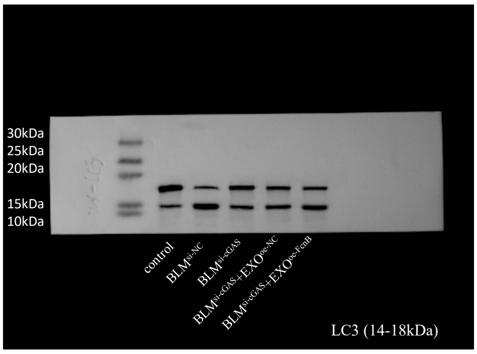

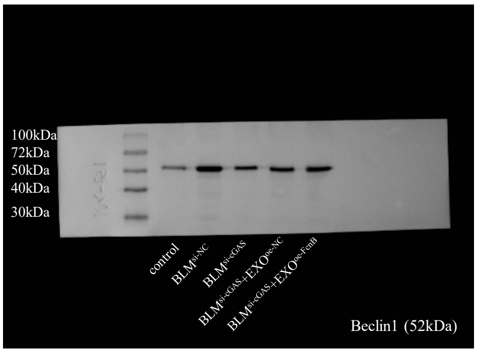

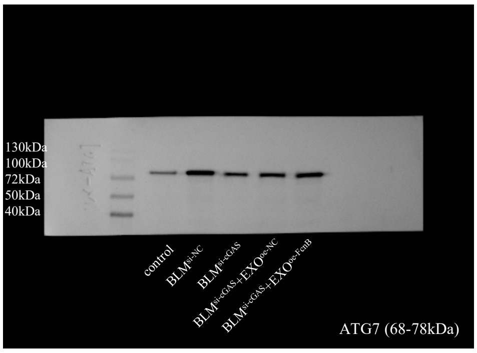

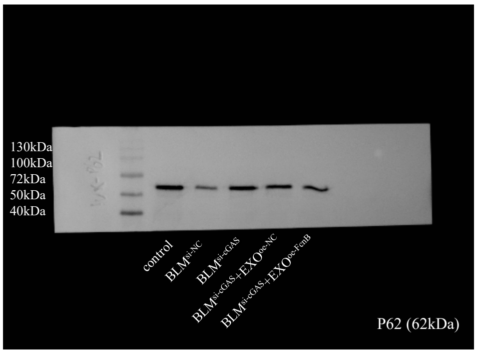

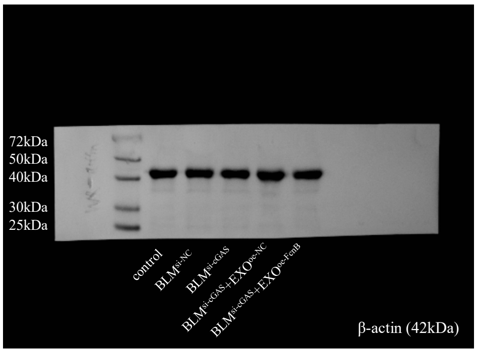

Figure 5H

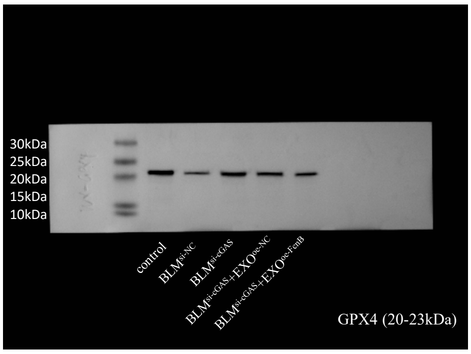

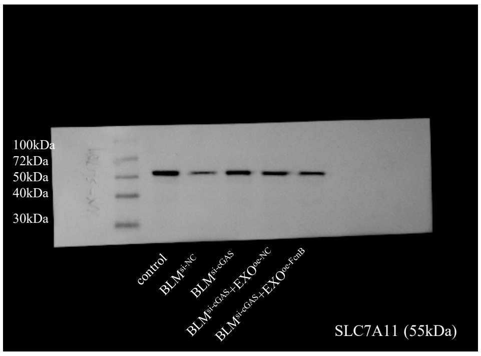

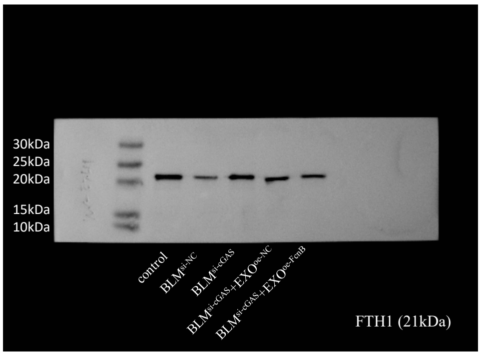

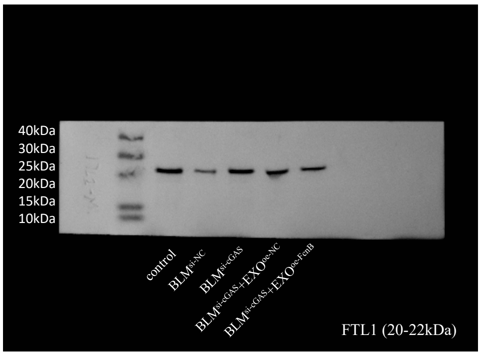

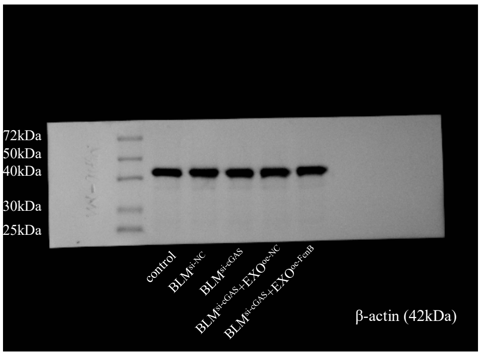

Figure 5I

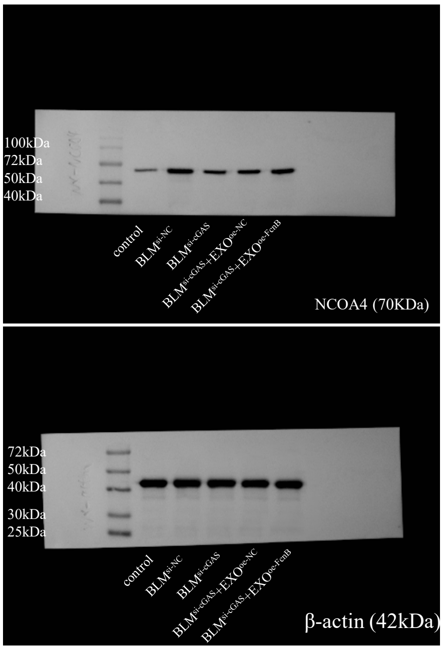

Figure 6B

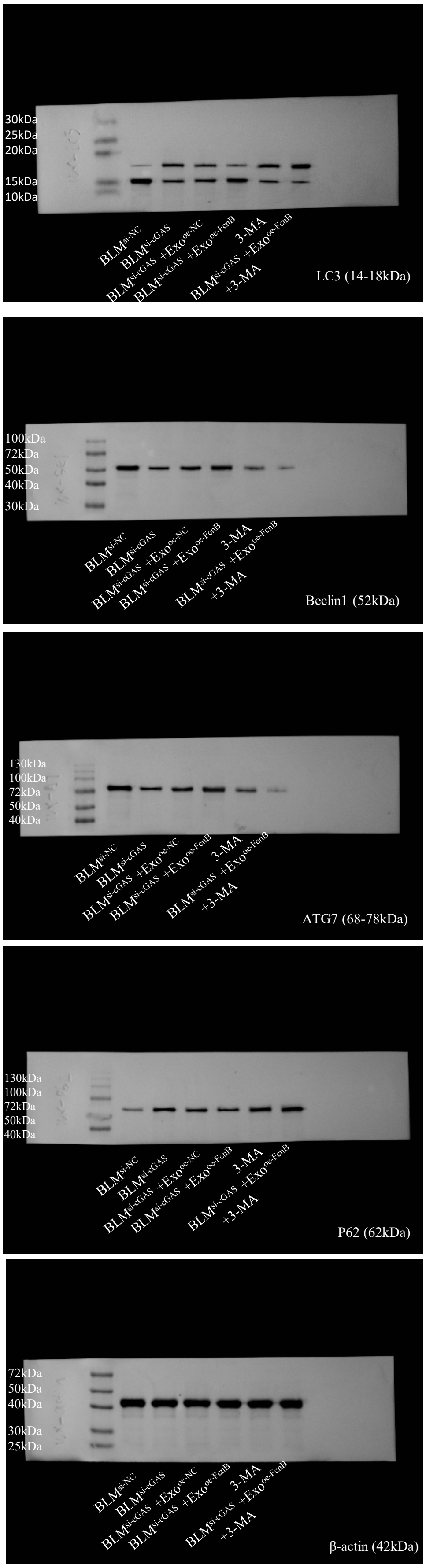

Figure 6H

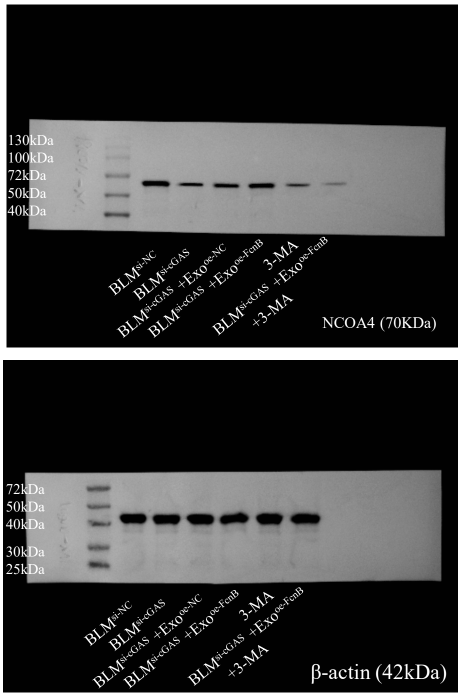

Figure 6G

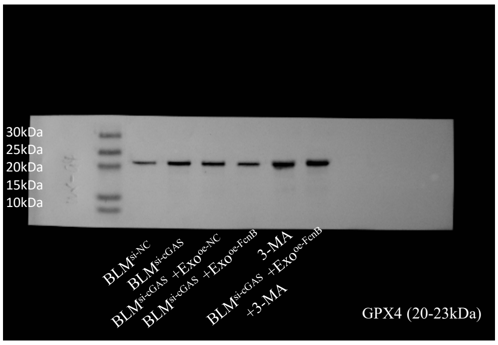

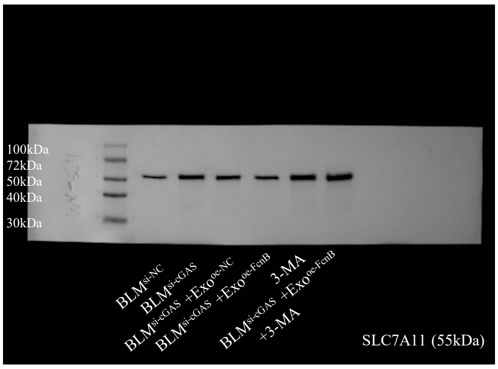

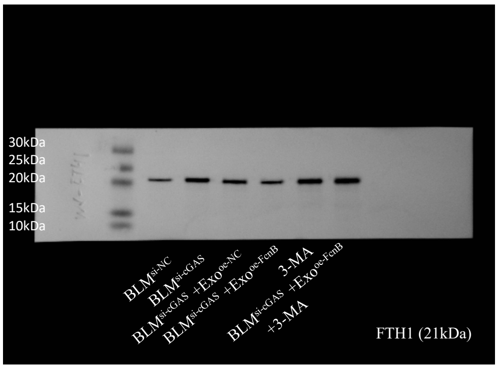

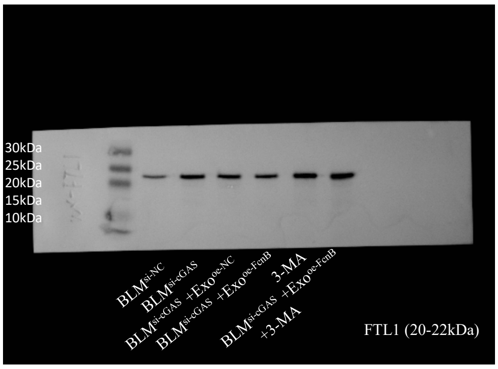

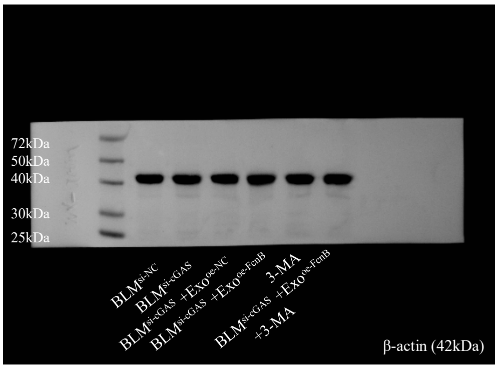

Figure 7E

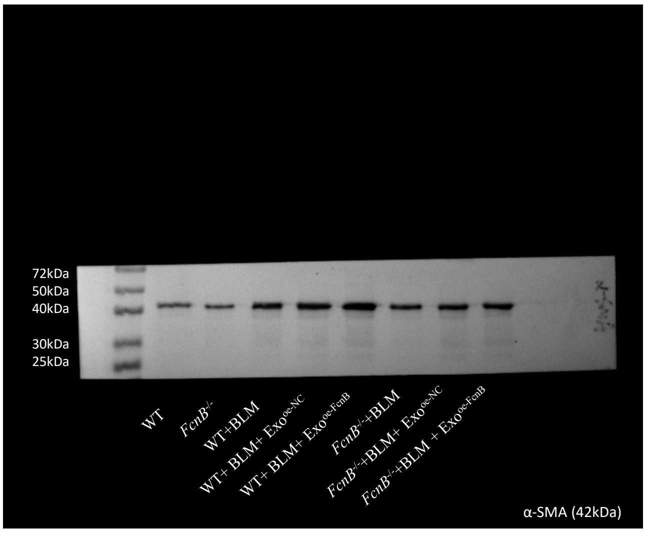

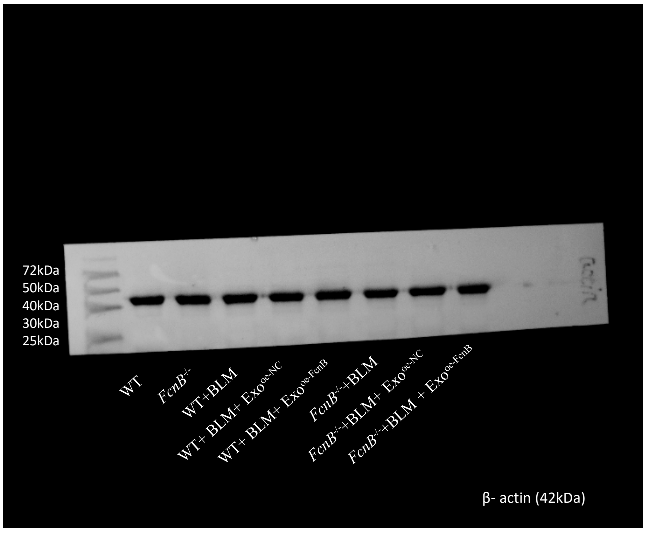

Figure 8B

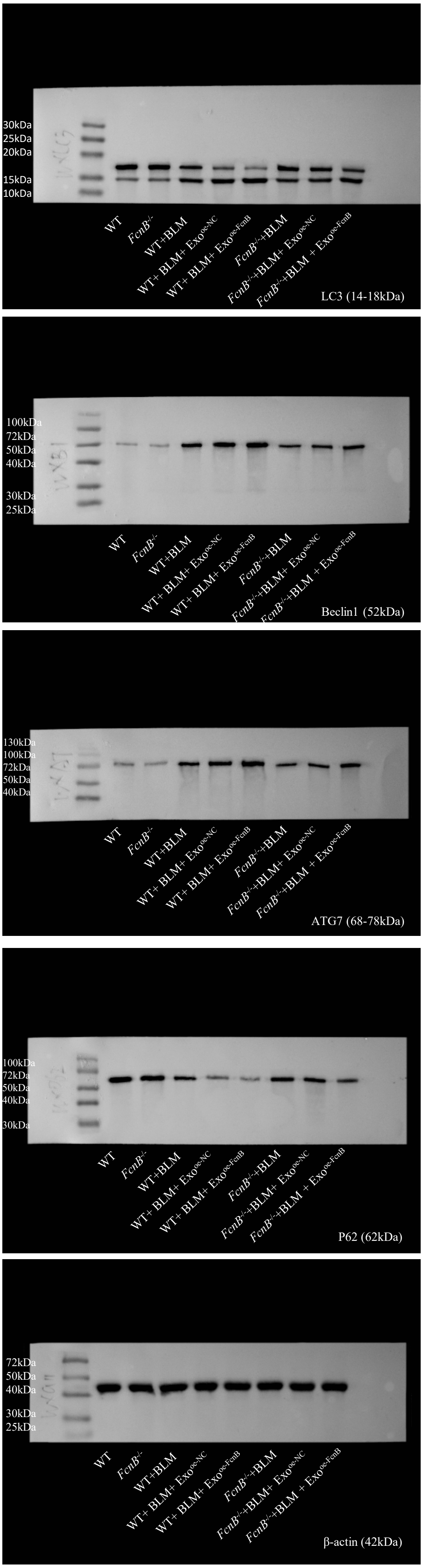

Figure 8F

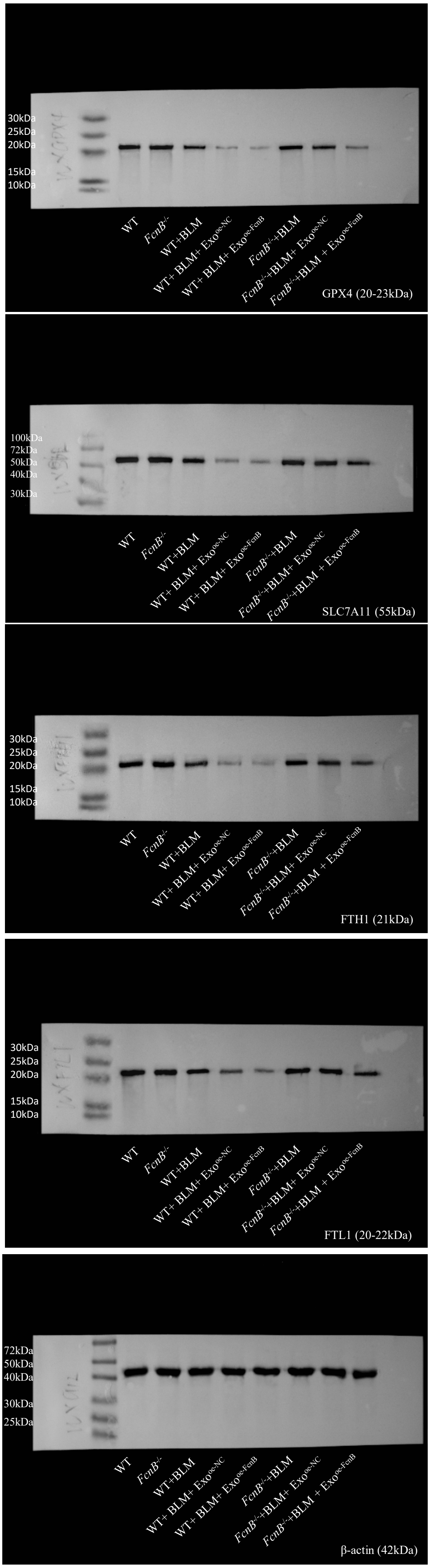

Figure 8F

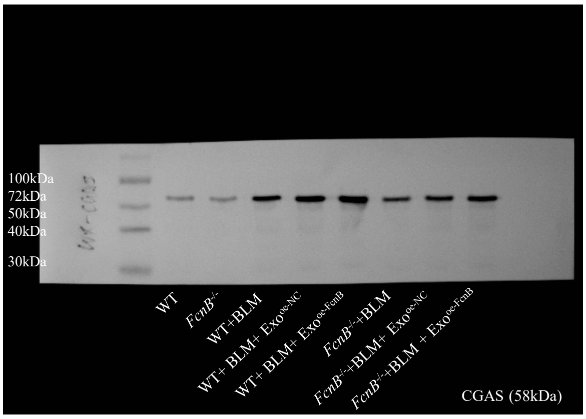

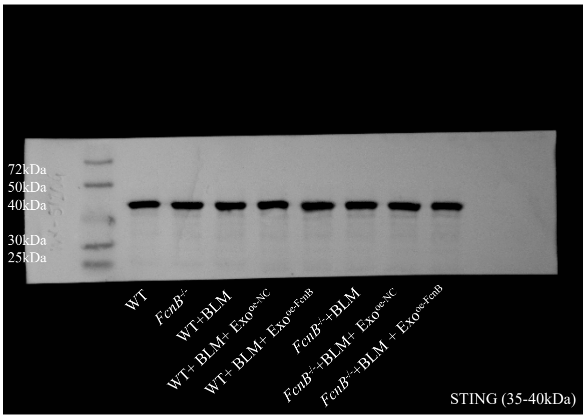

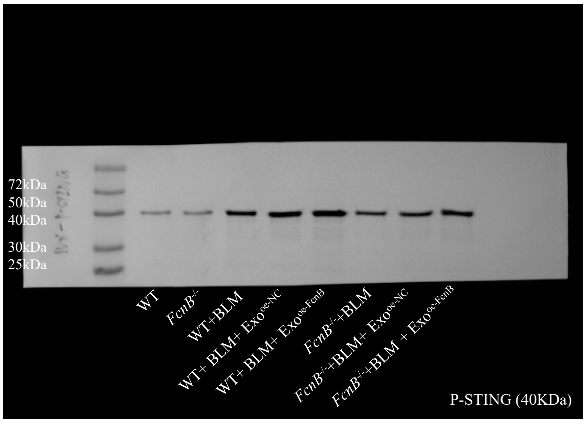

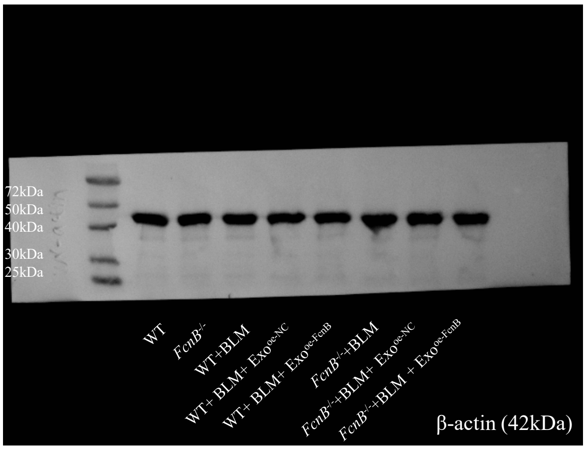

Figure 8G

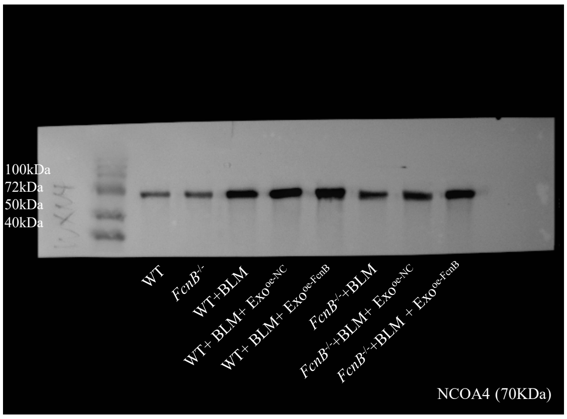

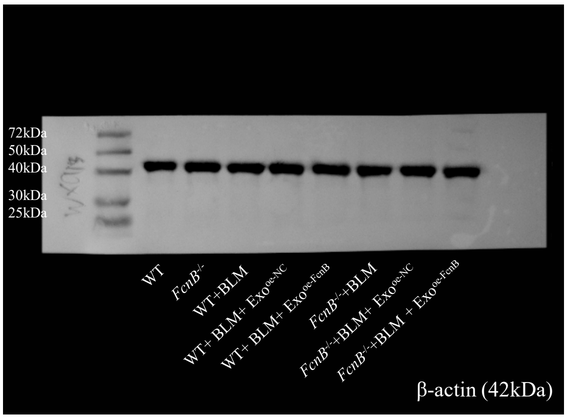

Supplement: Supplementary file 2 — Original Data File [file 41419_2023_6104_MOESM2_ESM.pdf]
